# FUS/TLS acts as an aggregation-dependent modifier of polyglutamine disease model mice

**DOI:** 10.1038/srep35236

**Published:** 2016-10-14

**Authors:** Yoshihiro Kino, Chika Washizu, Masaru Kurosawa, Mizuki Yamada, Hiroshi Doi, Toru Takumi, Hiroaki Adachi, Masahisa Katsuno, Gen Sobue, Geoffrey G. Hicks, Nobutaka Hattori, Tomomi Shimogori, Nobuyuki Nukina

**Affiliations:** 1 CREST(Core Research for Evolutionary Science and Technology), JST, Saitama, Japan; 2 Department of Neuroscience for Neurodegenerative Disorders, Juntendo University Graduate School of Medicine, Tokyo, Japan; 3 Laboratory for Structural Neuropathology , Brain Science Institute, RIKEN, Saitama, Japan; 4 Laboratory for Molecular Mechanisms of Thalamus Development, Brain Science Institute, RIKEN, Saitama, Japan; 5 Department of Bioinformatics and Molecular Neuropathology, Meiji Pharmaceutical University, Tokyo, Japan; 6 Department of Clinical Neurology and Stroke Medicine, Graduate School of Medicine, Yokohama City University, Yokohama, Japan; 7 Laboratory for Mental Biology, Brain Science Institute, RIKEN, Saitama, Japan; 8 Graduate School of Biomedical Sciences, Hiroshima University, Hiroshima, Japan; 9 Department of Neurology, Nagoya University Graduate School of Medicine, Nagoya, Japan; 10 Manitoba Institute of Cell Biology, University of Manitoba, Winnipeg, Canada; 11 Laboratory of Structural Neuropathology, Doshisha University Graduate School of Brain Science, Kyoto, Japan

## Abstract

FUS/TLS is an RNA/DNA-binding protein associated with neurodegenerative diseases including amyotrophic lateral sclerosis and frontotemporal lobar degeneration. Previously, we found that a prion-like domain in the N-terminus of FUS/TLS mediates co-aggregation between FUS/TLS and mutant huntingtin, the gene product of Huntington’s disease (HD). Here, we show that heterozygous knockout of FUS/TLS worsened the phenotypes of model mice of (HD, but not spinal and bulbar muscular atrophy (SBMA). This difference was correlated with the degree of pathological association between disease proteins and FUS/TLS. Co-aggregation between FUS/TLS and mutant huntingtin resulted in the depletion of free FUS/TLS protein in HD mice that was detected as a monomer in SDS-PAGE analysis. Recently, we found that FUS/TLS paralogs, TAF15 and EWS, were up-regulated in homozygous FUS/TLS knockout mice. These two proteins were up-regulated in both HD and FUS/TLS heterozygote mice, and were further elevated in HD-TLS^+/−^ double mutant mice, consistent with the functional impairment of FUS/TLS. These results suggest that FUS/TLS sequestration by co-aggregation is a rate-limiting factor of disease phenotypes of HD and that inclusions may have an adverse aspect, rather than being simply benign or protective. In addition, our results highlight inclusions as repositories of potential modifiers of neurodegeneration.

FUS/TLS is an RNA/DNA-binding protein associated with neurodegenerative diseases. Mutations in *FUS/TLS* cause familial amyotrophic lateral sclerosis (ALS)^1^,^2^. Inclusions containing this protein are observed in people with sporadic ALS and a subset of individuals with frontotemporal lobar degeneration (FTLD)^3^,^4^. The pathogenic mechanisms of these diseases remain unclear. Transgenic mice overexpressing FUS/TLS recapitulate ALS-like phenotypes^5^,^6^,^7^. While inbred FUS/TLS knockout mice show early postnatal lethality, outbred knockout mice manifest behavioral and histological abnormalities distinct from ALS but potentially relevant to FTLD^8^,^9^. FUS/TLS regulates RNA metabolisms, which include transcription and post-transcriptional processing related to neuronal functions^10^,^11^,^12^,^13^. The N-terminal region of FUS/TLS is a low complexity region enriched in Gln, Ser, Tyr, and Gly residues and is known as a prion-like domain^14^. This domain forms reversible amyloid-like assemblies that are involved in the formation of RNA-containing granules and transcriptional activation^15^,^16^.

In addition to its relationship to ALS and FTLD, FUS/TLS was found to be a major component of mutant huntingtin (Htt) aggregates in a cellular model of Huntington’s disease (HD)^17^. The interaction between FUS/TLS and mutant Htt is mediated by the prion-like domain of FUS/TLS^17^. HD is a fatal neurodegenerative disease primarily affecting the striatum and it involves movement, cognitive, and psychiatric dysfunction. HD is one of several polyglutamine (polyQ) diseases that are caused by the genetic expansion of a CAG repeat encoding polyQ^18^. Lines of evidence have emphasized the importance of an N-terminal fragment of Htt, the mutated protein in HD. Transgenic mice of the Htt exon 1 region, the R6/2 strain, exhibit HD-like phenotypes that are shared with HD knock-in model mice^19^,^20^. Moreover, these two mouse models show transcriptome changes similar to those in human HD^21^. N-terminal fragments of Htt are produced in both human HD and knock-in mice and have a greater aggregation propensity compared to the full-length protein^22^,^23^,^24^. Although intranuclear inclusions are a pathological hallmark of HD, cellular studies suggest that inclusions reflect protective responses while misfolded monomers are the putative toxic species^25^,^26^,^27^. In contrast, animal studies revealed reduction of monomeric Htt together with a concomitant increase in aggregates as well as inclusions during disease progression^19^,^28^,^29^.

In attempts to determine key molecules in HD, we identified component proteins of Htt aggregates, including FUS/TLS, EWS, TAF15, UBQLN2, and SQSTM1/p62, from a cellular model of HD^17^,^30^,^31^. Remarkably, mutations or pathological changes associated with ALS and FTLD were subsequently identified in these component proteins^1^,^2^,^14^,^32^,^33^. These results may indicate broader roles of these ALS/FTLD proteins in multiple neurodegenerative diseases. While the roles of FUS/TLS and its paralogs (EWS and TAF15) remain elusive, UBQLN2 and SQSTM1/p62 are involved in protein quality control, including autophagy, which may counteract the accumulation of mutant proteins. Two other ALS-linked proteins, OPTN and VCP, have been detected in the inclusions of polyQ diseases^34^,^35^,^36^. Thus, polyQ inclusions appear to be a repository of proteins related to multiple neurodegenerative diseases, implying a potential link between polyQ disease and ALS/FTLD. Indeed, the CAG repeat lengths of *ATXN2*, a polyQ disease gene, act as a risk factor for ALS^37^.

In this study, we identified component proteins of mutant Htt aggregates isolated from HD model mice. Consistent with our previous results, we identified FUS/TLS as a tightly bound component of the Htt aggregates *in vivo*. To elucidate the role of FUS/TLS in polyQ diseases, we crossed FUS/TLS heterozygous mice with transgenic (TG) model mice of both HD and spinal and bulbar muscular atrophy (SBMA), a motor neuron disease caused by a CAG/polyQ expansion of the androgen receptor (AR). We anticipated that FUS/TLS, as a causative gene of ALS, might modify another motor neuron disease. Although HD and SBMA impair distinct central nervous system (CNS) regions, comparison of these models would reveal their similarities and differences as well as the role of FUS/TLS in the disease conditions. We found that FUS/TLS acts as a modifier of HD, but not SBMA. This difference was correlated with reduction in the functional FUS/TLS protein through pathological co-aggregation with disease proteins. These results demonstrate a disease-specific role of FUS/TLS and highlight inclusions/aggregates as a reservoir of key proteins involved in neurodegenerative disorders.

## Results

### FUS/TLS binds to expanded polyglutamine proteins *in vivo*

**Figure 1:**
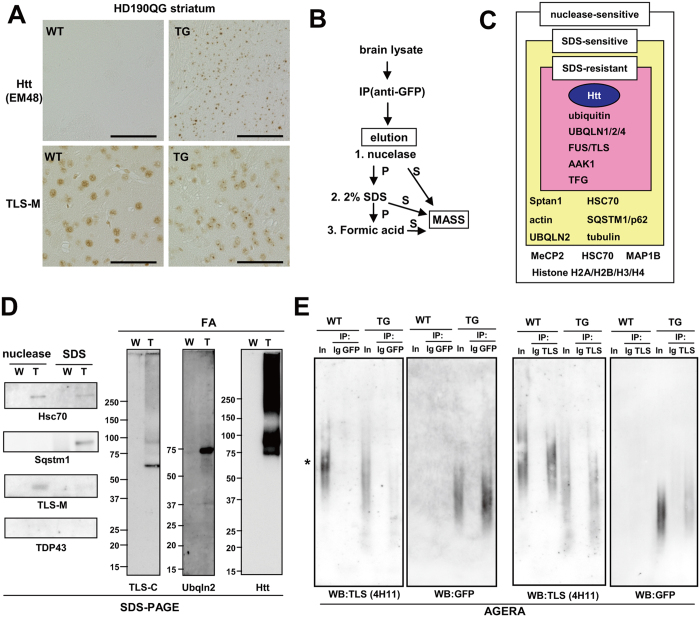
FUS/TLS binds to mutant huntingtin *in vivo*. (**A**) Protein distribution of Htt-EGFP and FUS/TLS in HD190QG and control animals at 24 weeks. Scale bar: 50 μm. (**B**) Procedure of immunoisolation of mutant Htt aggregates from HD190QG brain. S: supernatant, P: pellet. (**C**) Summary of mass analysis using eluted fractions from GFP immunoprecipitates. (**D**) Western blot analysis of eluted fractions of GFP immunoprecipitates. (**E**) AGERA of immunoprecipitates of anti-GFP and anti-TLS-M eluted by 2% SDS and 1M DTT. Membranes were stained with anti-GFP or anti-TLS (4H11).

To characterize mutant Htt aggregates *in vivo*, we fractioned brain lysates of transgenic mice (HD190QG) expressing exon 1 of mutant Htt fused with enhanced green fluorescent protein (EGFP)^38^. As in other HD model mice, we observed inclusions of mutant Htt in the striatum at 24 weeks of age (Fig. 1A, top panels). EGFP enabled us to monitor the mutant protein by its fluorescence, which was at least partially resistant to 2% SDS (Supplementary Fig. S1A). To visualize the high molecular weight aggregates, protein fractions were mixed with loading buffer containing 2% SDS without boiling and subjected to agarose gel electrophoresis for resolving aggregates (AGERA), which can resolve high-molecular-weight protein complexes^39^. Htt-EGFP was mostly detected as aggregates in high-salt nuclear extract fraction (NE) and SDS-containing fractions (S2, S3) (Supplementary Fig. S1B). To identify proteins associated with mutant Htt, the Htt complex was immunopurified with magnetic beads conjugated with anti-GFP antibody and sequentially eluted by nuclease, 2% SDS, and formic acid (Fig. 1B). The components of the Htt complex were identified by mass analysis. We identified UBQLN proteins, FUS/TLS, and AAK1 as major components in SDS-resistant fractions with high scores (Fig. 1C and Supplementary Table S1). FUS/TLS was detected in nuclear inclusions in HD190QG as in R6/2 (Fig. 1A and Supplementary Fig. S2A), in an age-dependent manner (Supplementary Fig. S2B). AGERA analysis revealed that FUS/TLS co-migrated with SDS-resistant Htt aggregates (Supplementary Fig. S1C). This co-migration was specific, because another RNA-binding protein, TDP-43, was hardly observed in the high-molecular-weight smear (Supplementary Fig. S1C). Among the three ubiquilin proteins identified, UBQLN2 was detected at a higher score than the others (Supplementary Table S1). Western analysis of the sequentially eluted samples (Fig. 1B) revealed that both FUS/TLS and UBQLN2 were detected in the formic acid-solubilized fraction (Fig. 1D). We also found Sqstm1/p62 as an SDS-sensitive component (Fig. 1C,D). Remarkably, FUS/TLS, but not TDP-43, was also detected in the nuclease-sensitive fraction, suggesting that this protein is associated with the Htt complex through nucleic acids (Fig. 1D). Finally, we tested the association between FUS/TLS and mutant Htt. Immunoprecipitates of anti-TLS or anti-GFP antibody was eluted with 2% SDS and resolved by AGERA to specifically detect FUS/TLS in SDS-resistant complexes. In the TG lysate, FUS/TLS was co-precipitated using anti-GFP, and GFP-containing aggregates were co-precipitated using anti-TLS (Fig. 1E), demonstrating that FUS/TLS is incorporated into Htt aggregates through SDS-resistant tight binding. Notably, we observed SDS-resistant high molecular weight species of FUS/TLS even from wild type (WT) lysate in the AGERA analysis, which migrated slower than those in TG complex (Fig. 1E, asterisk). Collectively, these results demonstrate that FUS/TLS is specifically incorporated into the SDS-resistant high-molecular-weight complex of mutant Htt *in vivo*. In addition, nucleic acids were found to be components of Htt aggregates that indirectly sequester some fraction of FUS/TLS into the aggregates, revealing biochemically multi-tiered architecture of Htt aggregates.

### Heterozygous deletion of FUS/TLS differentially affects polyQ disease model mice

**Figure 2:**
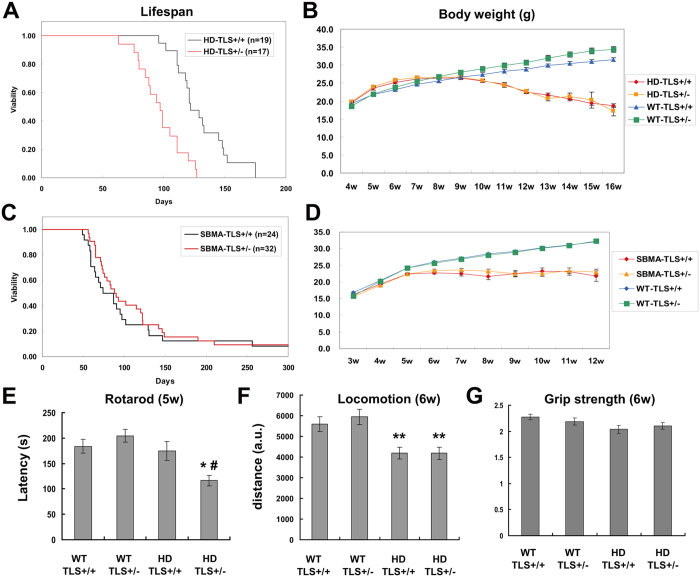
The effects of heterozygous deletion of FUS/TLS on polyQ disease model mice. (**A**,**C**) Kaplan-Meier analysis of HD-TLS (**A**) or SBMA-TLS (**C**) animals. The numbers of animals used are indicated in insets. FUS/TLS depletion significantly shortened the life span of HD (P < 0.0001) but not SBMA (P = 0.46) model mice in log-rank tests. (**B**,**D**) body weight changes in HD-TLS (**B**) and SBMA-TLS (**D**) animals. (**E**) Rotarod performance of HD-TLS mice at 5 weeks. Average latencies to fall are shown (n = 14 ~ 16). (**F**) Total distances of HD-TLS mice in an open field test (n = 14 ~ 16). (**G**) Grip strength of HD-TLS mice at 6 weeks of age (n = 12 ~ 15). *P < 0.05 in comparison with NT-WT, ^#^P < 0.05 in comparison with HD-WT by ANOVA followed by Tukey’s test. Error bars: SEM.

We next investigated the consequences of the association of FUS/TLS with polyQ complexes. The function of FUS/TLS might be reduced due to sequestration. Alternatively, the Htt-bound FUS/TLS might have adverse effects, as a kind of toxic gain of function. Additionally, it is also possible that FUS/TLS-polyQ association has no effect on the disease. To examine whether FUS/TLS acts as a modifier of polyQ disease phenotypes, we crossed TLS^+/−^ mice carrying a null mutation of *Fus/Tls*^8^ with transgenic model mice of HD and SBMA (Supplementary Fig. S3A). For HD model mice, we used the R6/2 strain that carries exon 1 of mutant Htt. For SBMA, we used mice carrying full-length mutant AR protein^40^. Importantly, both R6/2 and SBMA mice showed a similar age of disease onset (6 ~ 8 weeks) and life span (~20 weeks). The body weight, life span, and motor performance of SBMA-TLS^+/−^ mice were indistinguishable from those of SBMA-TLS^+/+^ mice (Fig. 2C,D, Supplementary Fig. S3B,C). In contrast, HD-TLS^+/−^ mice showed a markedly shortened life span compared to HD-TLS^+/+^ mice by 23.6% (Fig. 2A). Body weight was not different (Fig. 2B). A Rotarod test revealed significantly worsened motor performance of HD-TLS^+/−^ mice compared to HD-TLS^+/+^ mice at an early disease stage (Fig. 2E), whereas locomotion and grip strength of these mice were comparable (Fig. 2F,G). Motor performance and body weight of non-transgenic TLS^+/−^ mice were equivalent to those of wild-type mice (Fig. 2B,E). Additionally, the life span of inbred TLS^+/−^ mice was not significantly altered compared to TLS^+/+^ mice (median >2 years; P = 0.56, log-rank test; n = 22 for TLS^+/+^; n = 20 for TLS^+/−^). Therefore, FUS/TLS heterozygosity differentially affects the phenotypes of polyQ disease models.

### FUS/TLS protein is differentially sequestered in HD and SBMA model mice

**Figure 3:**
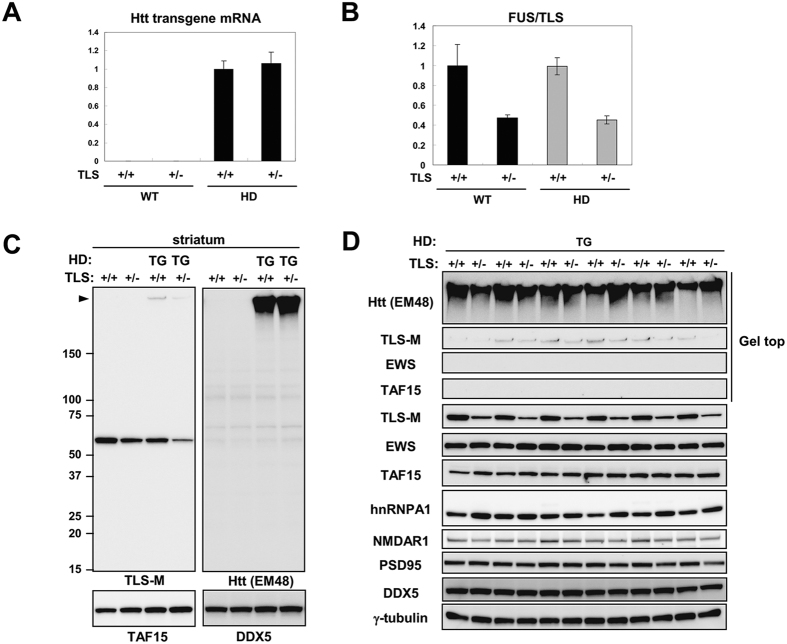
Protein expression in HD and SBMA model mice with or without FUS/TLS depletion. (**A**) qPCR analysis of transgene mRNA expression in the striatum of crossed animals. Error bars represent SEM (n = 3). (**B**) qPCR analysis of FUS/TLS expression in the striatum of HD-TLS crossed animals (n = 3). (**C**) Western blot analysis of HD-TLS animals. Genotypes and antibodies are indicated. (**D**) Western blot analysis of the striatum of HD-TLS^+/+^ and HD-TLS^+/−^ animals (n = 6). Genotypes and antibodies are indicated. Arrowhead in (**C**) indicates gel top.

Hereafter, we mainly focused on the striatum of HD mice and the spinal cord of SBMA mice, because these regions are the most susceptible in each of these diseases. As a reduced level of FUS/TLS worsened the phenotype of HD model mice, we considered two possibilities: (i) FUS/TLS regulates mutant Htt protein expression and/or aggregation as we observed *in vitro* previously^17^; or (ii) loss of FUS/TLS function is critical for disease progression. Htt transgene expression was not affected by FUS/TLS heterozygous deletion in HD-TLS double-mutant mice (Fig. 3A). The mRNA levels of FUS/TLS in both WT-TLS^+/−^ and TG-TLS^+/−^ mice was about 50% of WT-TLS^+/+^ mice (Fig. 3B). In HD mice, the amount of mutant Htt protein and its aggregates as not altered by FUS/TLS heterozygosity (Fig. 3C,D). Thus, it was unlikely that the exacerbated phenotypes caused by FUS/TLS heterozygosity were mediated by altered expression or aggregation of mutant Htt. In contrast, monomeric FUS/TLS protein in HD-TLS^+/−^ mice was markedly reduced compared to other genotypes; the level of monomeric FUS/TLS in WT-TLS^+/−^ mice was 48 ± 8.0% of WT-TLS^+/+^ mice, whereas that in HD-TLS^+/−^ mice was 28 ± 1.2% of WT-TLS^+/+^ mice (mean ± SEM, n = 4). The reduction of monomeric FUS/TLS could be due to incorporation of FUS/TLS Supplementary Fig. S4A,B). Such incorporation was not observed for EWS or TAF15, suggesting a higher propensity of FUS/TLS for co-aggregation with Htt (Fig. 3D).

**Figure 4:**
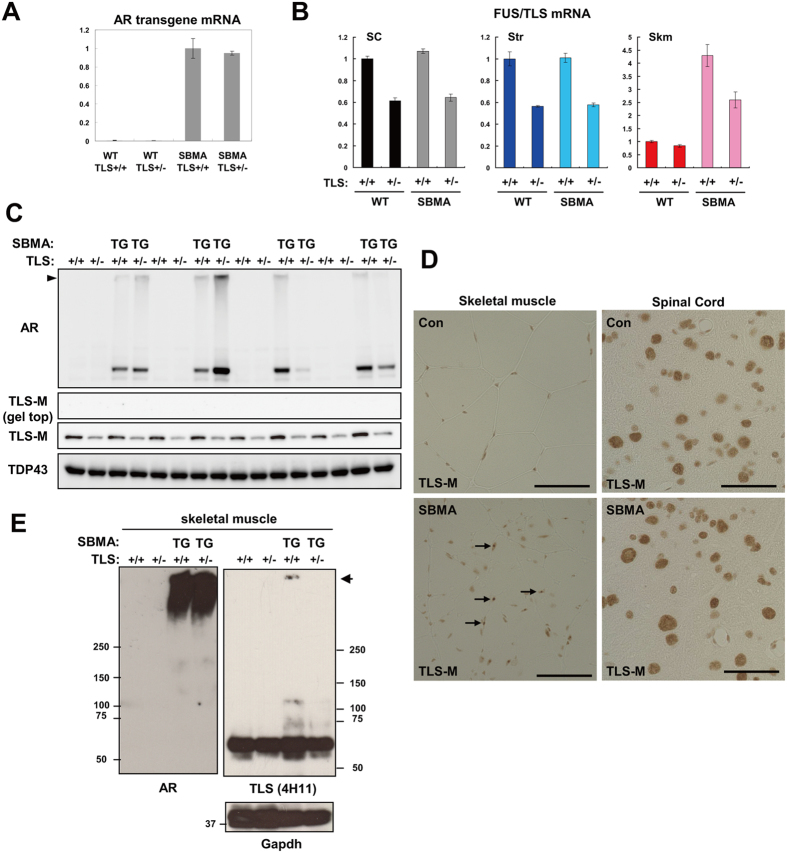
Protein expression in HD and SBMA model mice with or without FUS/TLS depletion. (**A**) qPCR analysis of transgene mRNA expression in crossed animals. Error bars represent SEM (n = 4). (**B**) qPCR analysis of FUS/TLS expression in the spinal cord (SC), striatum (Str), and skeletal muscle (Skm) of SBMA-TLS crossed animals (n = 4). (**C**) Western blot analysis of the spinal cord of SBMA-TLS mice (n = 4). N-20 antibody was used for AR. Arrow head indicates gel top. (**D**) Immunohistochemical analysis of SBMA and control mice. Spinal cord and skeletal muscle sections were stained with TLS-M antibody. Arrows indicate inclusions. Scale bar = 50 μm. (**E**) Western blot analysis of the skeletal muscle of SBMA-TLS mice. Arrow indicates gel top.

In the spinal cord of SBMA-TLS mice, FUS/TLS heterozygosity did not alter the mRNA level of AR transgene (Fig. 4A). There was a large variability in AR expression among animals and the effect of FUS/TLS heterozygosity was not obvious (Fig. 4C). In contrast to the HD-TLS animals, we hardly detected FUS/TLS protein at the gel top in the case of SBMA-TLS animals (Fig. 4C). In contrast to mutant Htt in HD mice that was mostly detected at the gel top (Fig. 3C), the majority of AR was detected as a monomer (Fig. 4C). The amount of monomeric FUS/TLS in SBMA-TLS^+/−^ (51 ± 1.0% of WT-TLS^+/+^, n = 4) was not decreased compared to that in WT-TLS^+/−^ (44 ± 2.0% of WT-TLS^+/+^, Fig. 4C). In immunohistochemical analysis, FUS/TLS was mainly localized in the nuclei of motor neurons and other cells in the spinal cord of wild-type animals (Fig. 4D). FUS/TLS showed a similar nuclear staining in SBMA-TLS^+/+^ mice (Fig. 4D). Interestingly, skeletal muscle of SBMA-TLS^+/+^ mice showed occasional nuclear aggregation of FUS/TLS (Fig. 4D). FUS/TLS colocalized with these inclusions, but was still observed in the intranuclear regions outside of the aggregates (Supplementary Fig. S5A). Mutant AR protein was mostly detected as aggregates in the SBMA skeletal muscle (Fig. 4E). Consistent with immunohistochemical analysis, we detected aggregation of FUS/TLS in SBMA-TLS^+/+^ mice (Fig. 4E). Importantly, however, monomeric FUS/TLS in the skeletal muscle was not reduced in SBMA-TLS^+/−^ mice compared to WT-TLS^+/−^ mice (both ~90% of WT-TLS^+/+^ mice, Fig. 4E). We noticed that both mRNA and protein levels of FUS/TLS was relatively high in the skeletal muscle of WT-TLS^+/−^ mice (84% and 89%, respectively, of WT-TLS^+/+^ mice, Fig. 4B,E). Moreover, mRNA levels of FUS/TLS were elevated in the skeletal muscle (>2-fold), but not spinal cord or striatum, of SBMA-TLS^+/+^ and SBMA-TLS^+/−^ mice (Fig. 4B). Thus, FUS/TLS expression in the skeletal muscle was kept at near-normal levels in FUS/TLS heterozygotes and responded to mutant AR expression differently from that in spinal cord and striatum. In Summary, FUS/TLS depletion was not strongly elicited in SBMA mice, because AR was not markedly aggregated in the spinal cord while FUS/TLS level was elevated in the skeletal muscle.

**Figure 5:**
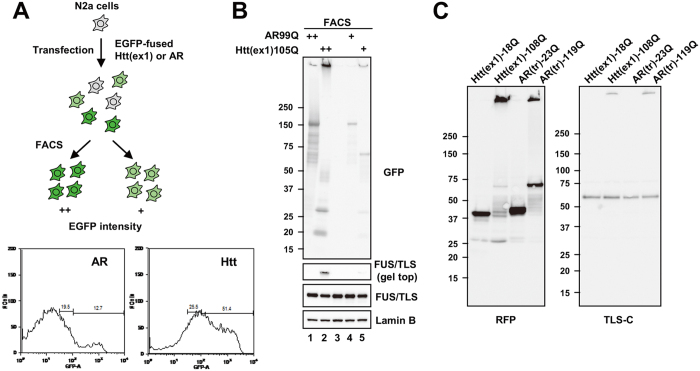
Co-aggregation of FUS/TLS is dependent on protein truncation. (**A**) Comparison of aggregation properties of mutant Htt exon 1 and full-length AR fused with EGFP. To prepare cells with comparable expression levels of Htt or AR, transfected Neuro2a cells were selected according to their intensities of EGFP fluorescence using FACS. Two classes of cells were prepared; high expression (++) and moderate expression (+). The same number of cells were collected for each condition. Bottom panels show the fluorescent profiles of transfected cells used for cell sorting. (**B**) Western analysis of total lysates from expression-matched cells with Htt or AR. Htt showed larger fraction of aggregates as well as co-aggregation of endogenous FUS/TLS than AR. Furthermore, even though the protein level of Htt(+) was smaller than that of AR(++), the former showed higher degree of aggregation of itself as well as FUS/TLS (lanes 1 and 5). See also Supplementary Fig. S4D for quantification. (**C**) Truncated mutant AR showed aggregation properties similar to Htt exon 1. An N-terminal fragment (amino acids 1–132) of AR or Htt exon 1 was expressed as a fusion with RFP in N2a cells. The total lysates were used for Western analysis.

Finally, we directly compared aggregation properties of Htt exon 1 [Htt(ex1)105Q] and full-length AR (AR99Q) using expression level-matched cells (Fig. 5A). Htt(ex1)105Q was more prone to aggregate than AR99Q and induced stronger FUS/TLS co-aggregation (Fig. 5B). Importantly, the higher-amount AR99Q fraction (++, lane 1) contained gel top aggregates comparable to the lower-amount Htt(ex1)105Q fraction (+, lane 5). However, co-aggregated FUS/TLS at the gel top was only detectable for Htt(ex1)105Q (lane 5, Fig. 5B and Supplementary Fig. S4D), suggesting that the aggregates of Htt(ex1)105Q have higher affinity for FUS/TLS than those of AR99Q. We also found that truncated AR [AR(tr)119Q] induced co-aggregation of FUS/TLS similarly to Htt exon 1 (Fig. 5C), indicating that protein truncation facilitates co-aggregation. We confirmed that FUS/TLS binds to the aggregates of truncated AR by immunoprecipitation (Supplementary Fig. S5B). In conclusion, the differential consequence of FUS/TLS heterozygosity in the polyQ disease model mice was correlated with the pathological co-aggregation and depletion of FUS/TLS.

### Gene expression changes in HD-TLS^+/−^ mice

**Figure 6:**
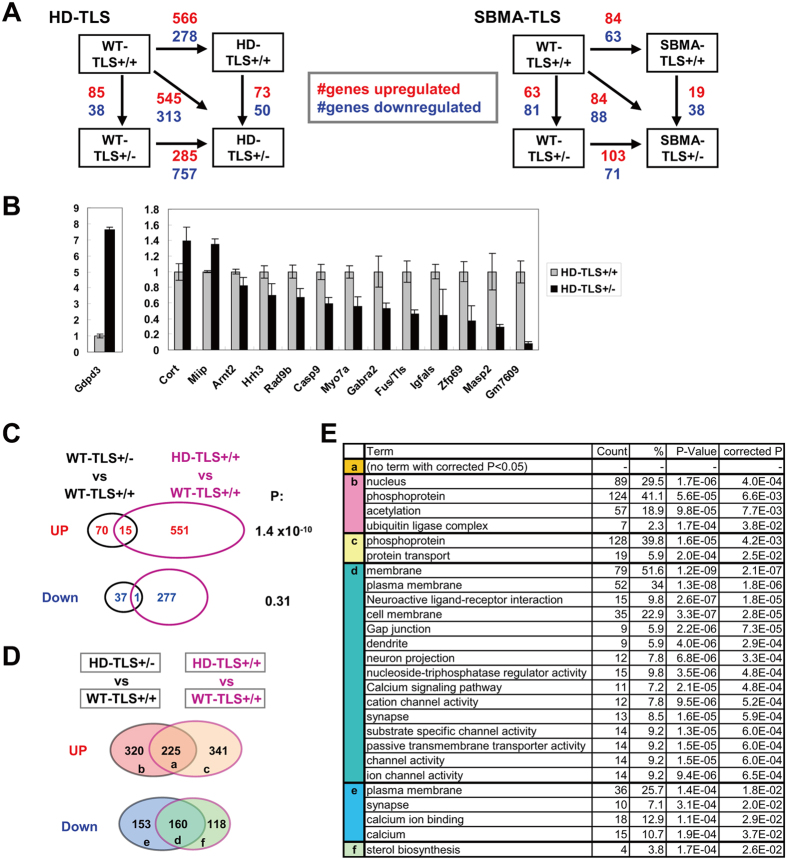
mRNA expression in HD model mice with FUS/TLS heterozygous depletion. (**A**) Gene expression analysis of HD-TLS (striatum) and SBMA-TLS (spinal cord) samples. (**B**) qPCR analysis of selected genes whose expression in the striatum was altered between HD-TLS^+/−^ and HD-TLS^+/+^ in ExonArray analysis. Mean ± SEM (n = 3). (**C**) Overlap of genes that are altered by FUS/TLS depletion and transgene expression. Venn diagrams indicate number of downregulated or upregulated genes in both WT-TLS^+/−^ and HD-TLS^+/+^ animals compared to WT-TLS^+/+^ (striatum). P-values of Fisher’s exact test are shown for the significance of overlap. (**D**) Comparison of genes altered between HD-TLS^+/−^ and WT-TLS^+/+^ and between HD-TLS^+/+^ and WT-TLS^+/+^ (striatum). Venn diagrams show number of genes. Lowercase letters indicate groups of genes in the diagram. Mean ± SEM (n = 3). (**E**) Gene ontology analysis of gene groups indicated in D using DAVID. Terms with a corrected P value <0.05 are shown. For group d, top 15 terms from 75 significant terms are shown.

The worsened phenotype of HD mice by FUS/TLS heterozygosity could be due to loss of function of FUS/TLS. Since FUS/TLS has been implicated in the regulation of transcription and post-transcriptional RNA processing, we analyzed the transcriptome changes of 8-week-old HD-TLS crossed mice using ExonArray, which probes gene expression at the resolution of each exon. First, we identified 123 genes (38 upregulated and 85 downregulated) whose expression was changed in non-transgenic WT-TLS^+/−^ mice compared to WT-TLS^+/+^ mice (Fig. 6A and Supplementary Table S2). This gene set represented genes altered by FUS/TLS heterozygous deletion. Second, we identified 123 genes (73 upregulated, 50 downregulated) differentially expressed in the striatum of HD mice with and without FUS/TLS heterozygous deletion (HD-TLS^+/−^ vs HD-TLS^+/+^), which may reflect the phenotypic differences between these genotypes. We confirmed the ExonArray results using qPCR (Fig. 6B). These included receptors for histamine and GABA (Hrh3 and Gabra2), which are involved in neuronal functions. Upregulation of cortistatin (Cort) was of interest as this neuropeptide can depress neuronal activities^41^. In the case of SBMA-TLS crossed mice, the number of genes altered by FUS/TLS heterozygosity (WT-TLS^+/−^ vs WT-TLS^+/+^) in the spinal cord was comparable to that in HD-TLS mice (Fig. 6A). In contrast, we detected a smaller number of genes altered by mutant protein expression (SBMA-TLS^+/+^ vs WT-TLS^+/+^) compared to HD, suggesting a greater impact of the mutant protein expression on the transcriptome in HD mice than in SBMA mice.

To estimate transgene-dependent gene expression changes that could be explained by FUS/TLS heterozygosity, we compared overlaps in the gene sets (Fig. 6C). Striatal genes upregulated by FUS/TLS depletion were well represented in genes altered by HD (15 overlaps out of 85 and 566 genes, P = 1.4 × 10^−10^, exact test), whereas downregulated genes were not (Fig. 6C). Thus, FUS/TLS may explain a small fraction (at most ~3%) of altered gene expression in HD model mice, though a majority of gene expression changes in HD must be explained by other factors. Similar analysis of SBMA-TLS mice revealed moderate overlaps in both up- and downregulated genes (Supplementary Fig. S6B). We also found an overlap of RNA processing altered by either HD or FUS/TLS heterozygosity (Supplementary Fig. S6C). FUS/TLS may explain roughly 5% of RNA processing changes in HD, suggesting that FUS/TLS abnormality mediates both transcriptional and post-transcriptional processing in the HD transcriptome. Finally, we compared genes altered between HD-TLS^+/+^ and WT-TLS^+/+^ to genes altered between HD-TLS^+/−^ and WT-TLS^+/+^ (Fig. 6D). These comparisons revealed larger differences between HD-TLS^+/+^ and HD-TLS^+/−^ (>300 upregulated and >100 downregulated genes, Fig. 6D) than the direct comparison of HD-TLS^+/−^ and HD-TLS^+/+^ (Fig. 6A). In gene ontology analysis, commonly upregulated genes (group a, in Fig. 6D) did not contain any specific gene ontology terms, whereas commonly downregulated genes (group d) were highly enriched in gene ontology terms related to neuronal functions known to be altered in HD. Remarkably, genes downregulated only in HD-TLS^+/−^ (group e) were also enriched in gene ontology terms associated with neuronal functions such as “synapse” and “calcium” that are shared by group d, whereas genes downregulated only in HD-TLS^+/+^ (group f) did not (Fig. 6E). Similar analysis of HD-TLS^+/−^ vs WT-TLS^+/−^ and HD-TLS^+/+^ vs WT-TLS^+/+^ revealed that FUS/TLS heterozygosity makes genes related to ion channels more sensitive to HD transgene expression (Supplementary Fig. S6A). These results indicate that misregulation of genes directly related to neuronal functions was extended or accelerated in HD-TLS^+/−^ mice compared to HD-TLS^+/+^ mice. In a similar analysis, SBMA-TLS crossed mice did not show enrichment of specific ontological terms in any of overlapping and non-overlapping gene groups, possibly due to the small number of genes altered in these mice. In conclusion, our results indicate that some gene expression changes in HD can be explained by reduced normal function of FUS/TLS (Fig. 6C), while FUS/TLS heterozygous deletion directly or indirectly affects gene expression specifically related to neuronal functions (Fig. 6D,E). This suggests that functional impairments of FUS/TLS mediate HD pathogenesis at the transcriptome level.

### TAF15 and EWS protein levels were up-regulated in HD-TLS^+/−^ mice

**Figure 7:**
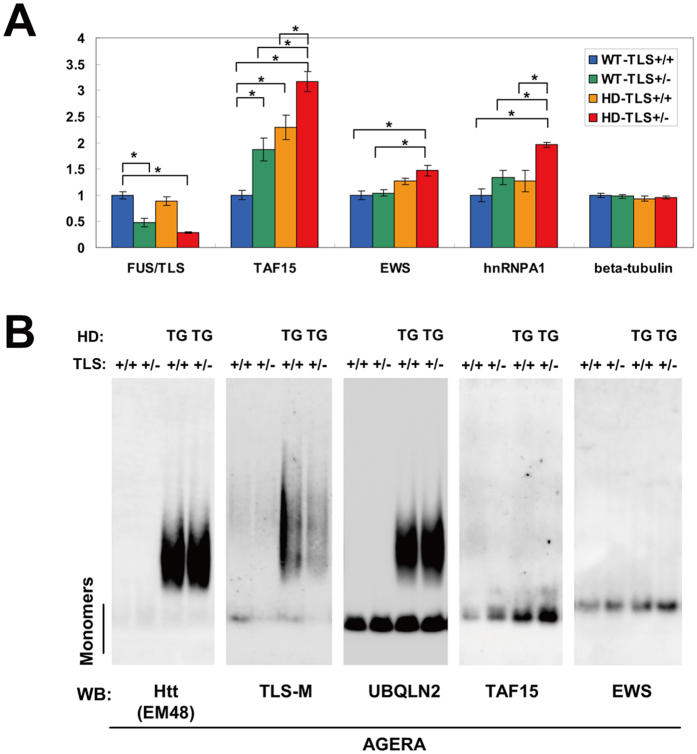
Protein levels of TAF15 and EWS were up-regulated in FUS/TLS-depleted mice. (**A**) Protein expression in the striatum of HD-TLS crossed mice (n = 4). For FUS/TLS, monomeric protein was quantified. DDX5 was used for normalization. *P < 0.05, ANOVA followed by Tukey’s post-hoc test. (**B**) AGERA analysis of HD-TLS crossed mice using antibodies indicated. FUS/TLS and UBQLN2 showed co-migration with mutant Htt, whereas TAF15 and EWS did not.

Recently, we found that TAF15 is upregulated in both FUS/TLS homozygote knockout mice and cultured cells depleted of FUS/TLS^9^. TAF15 protein levels were elevated in both WT-TLS^+/−^ and HD-TLS^+/+^, and even higher in HD-TLS^+/−^ mice (Figs 3D and 7A). Similarly, EWS and hnRNAPA1 are targets of FUS/TLS^9^ and were also elevated in HD-TLS^+/−^ mice (Figs 3D and 7A). Thus, the amount of monomeric FUS/TLS protein was inversely correlated with the amount of its targets in HD-TLS mice, consistent with the functional depletion of FUS/TLS in HD mice. AGERA analysis of HD-TLS mice revealed that FUS/TLS, but not TAF15 or EWS, was detected in the fraction of aggregates in HD mice, as with Htt and UBQLN2 (Fig. 7B). Therefore, even with elevated protein levels in HD-TLS^+/−^ mice, EWS and TAF15 did not co-migrate with aggregates in AGERA (Fig. 7B), pointing to the specificity of FUS/TLS co-aggregation in HD mice. We also examined AAK1, TFG, and Rad23b in AGERA but did not detect obvious changes in their patterns (Supplementary Fig. S4C). In conclusion, the sequestration of FUS/TLS into mutant Htt aggregates causes depletion of functional FUS/TLS protein, which results in the misregulated expression of its target genes.

## Discussion

In this study, we found that heterozygous deletion of FUS/TLS worsened the phenotypes of HD, but not SBMA, model mice. Using life span as a measure of disease phenotype, FUS/TLS can be regarded as a strong negative modifier acting at heterozygous deletion (Supplementary Fig. S3D). Interestingly, FUS/TLS interacts physically and functionally with two other negative modifier proteins, CBP and PGC-1α that regulate transcription^42^,^43^, suggesting a common molecular pathway involving these proteins in the HD transcriptome. We found that a small fraction of transcriptome changes in HD could be explained by partial loss of FUS/TLS function (Fig. 6C and Supplementary Fig. S6C). This is consistent with the involvement of multiple transcriptional regulators that are simultaneously affected by mutant Htt. It is possible that FUS/TLS regulates distinct sets of genes under normal and disease conditions. If this is the case, a larger fraction of changes in the transcriptome of polyQ diseases might be explained by FUS/TLS than has been described above. As FUS/TLS has been implicated in DNA damage response^7^,^44^, we do not exclude that the genetic effects of FUS/TLS heterozygosity were mediated by altered DNA damage responses.

Although caution should be taken when comparing HD and SBMA models using different tissues, we obtained contrasting results in the CNS regions most susceptible to each disease. In HD-TLS^+/−^ mice, the free fraction of FUS/TLS protein was decreased. Concomitantly, we observed altered protein expression of FUS/TLS target genes, TAF15, EWS, and hnRNPA1, indicating functional impairment of FUS/TLS in these mice (Figs 3D and 7A). Up-regulation of TAF15 and EWS can be regarded as a compensatory mechanism for the reduction of FUS/TLS, as these proteins share some RNA targets^45^. However, loss of FUS/TLS can not be fully compensated by EWS and TAF15, as demonstrated by the phenotypes of FUS/TLS knockout mice, in which both proteins were up-regulated^9^. In contrast to HD, we hardly observed co-aggregation of FUS/TLS with mutant AR in the spinal cord of SBMA mice. Consistently, normal FUS/TLS distribution has been reported in the spinal cord of an independent strain of SBMA mice^46^. In addition, we observed co-aggregation of FUS/TLS and mutant AR in the skeletal muscle of SBMA mice (Fig. 4E and Supplementary Fig. S5A). However, this co-aggregation was not enough to deplete the monomeric FUS/TLS fraction, reflecting (1) relatively high baseline level of FUS/TLS in the skeletal muscle of the heterozygous mice, (2) upregulation of FUS/TLS mRNA levels in SBMA muscle and (3) lower affinity of full-length AR for FUS/TLS compared to Htt(ex1) (Figs 4B and 5B). There results indicate that tissue- and disease-dependent factors determine whether co-aggregation is induced and whether it leads to dysfunction of proteins. Notably, genetic deletion of p62/SQSTM1, known as an important regulator of selective autophagy as well as a common component of inclusions in various neurodegenerative diseases, worsened the phenotypes of SBMA mice while ameliorated those of HD mice^47^,^48^. This also exemplifies disease-dependent effects of aggregate-associated proteins. In conclusion, FUS/TLS acts as a disease modifier depending on disease conditions, which was correlated with its co-aggregation with disease proteins and its functional depletion in the affected CNS regions.

We observed co-migration of mutant Htt, FUS/TLS and UBQLN2 as a wide-range smear in AGERA (Fig. 7B and Supplementary Fig. S1A). Importantly, the size distribution of this smear was similar to that observed in a previous study, where the aggregates are Htt oligomers of variable sizes^29^. Consistently, we previously found that FUS/TLS binds to not only Htt fibrils but also smaller amorphous aggregates that might be derived from oligomers *in vitro*^17^,^29^. Therefore, our results suggest that Htt oligomers bind to FUS/TLS and/or UBQLN2 and may mediate disease pathogenesis. Though we reported previously that FUS/TLS reduced Htt aggregation *in vitro*^17^, we did not observe changes in Htt aggregation upon FUS/TLS heterozygous depletion (Figs 3C and 7B). This might be because the amount of FUS/TLS or the degree of its depletion in the heterozygous mice was not enough to affect Htt aggregation *in vivo*. While FUS/TLS co-aggregates with mutant Htt through SDS-resistant tight interaction, some fraction of FUS/TLS binds indirectly through nucleic acids (Fig. 1D). Therefore FUS/TLS sequestration might be underestimated in SDS-PAGE, as its monomer may include a fraction of FUS/TLS that was sequestered into Htt aggregates through nucleic acids but was dissociated by the subsequent SDS treatment.

Previously, we found that the N-terminal low-complexity region of FUS/TLS mediates co-aggregation with mutant Htt^17^. The polyQ tract itself is a very low-complexity region. Thus, our results indicate deleterious consequences of the FUS/TLS-polyQ interaction through the low-complexity regions. While the N-terminus of FUS/TLS contributes to RNA metabolisms through its propensity to form reversible aggregates^15^,^49^, this property of FUS/TLS may be a source of the predisposition to persistent co-aggregation with mutant polyQ proteins and the formation of cytoplasmic inclusions in ALS/FTLD. Remarkably, mutations in RNA-binding proteins with low complexity regions (TDP-43, FUS/TLS, EWS, TAF15, hnRNPA2B1, and hnRNPA1) have been increasingly identified in ALS^14^,^33^,^50^,^51^. Together with the common abnormality of transcription in polyQ diseases^18^, these observations point to a fundamental link among RNA metabolism, protein aggregation, and neurodegenerative diseases mediated by low-complexity regions.

Inclusions/aggregates may accumulate proteins that cope with the aggregates as well as proteins that are susceptible to co-aggregation. Therefore, we propose that components of inclusions or aggregates must be regarded as a repository of potential disease-modifying proteins that can be involved in multiple neurological disorders. In this study, as a proof of concept, we verified that an ALS-linked protein, FUS/TLS, acts as a modifier of HD phenotypes in mice. Our results support that inclusions/aggregates have adverse aspects in the disease pathogenesis or progression, rather than they are simply benign or protective. It is likely that other components of Htt aggregates, especially UBQLNs and AAK1 (a polyQ-containing kinase), would be interesting candidates for modifiers of HD and possibly other diseases. Indeed, UBQLN2 mutations are known as a cause of ALS and ALS/dementia^32^. Its paralog, UBQLN1, has been reported as a protective modifier of R6/2 mice^52^. We also detected TFG in the Htt aggregates (Fig. 1C), a causative protein of a sensory and motor neuropathy with ALS-like pathology^53^. These observations indicate a pathological protein interaction network that underlies the etiology of multiple neurological disorders.

## Methods

### Ethic Statement

This study was carried out in accordance with the institutional guidelines and protocols approved by the RIKEN Animal Experiments Committee (approval number: H25-2-303(1)) and Genetic Recombinant Experiment Safety Committee (approval number: 2013-016(1)).

### Animals

All the experiments with mice were approved by the Animal Experiment Committee of the RIKEN Brain Science Institute. Animals were maintained on a 12 h light/dark cycle with access to food and water *ad libitum*. The temperature and humidity were maintained at 22–23 °C and 50–60%, respectively. Animal health was checked by the animal facility staffs five times per week. Heterozygote TLS knockout (TLS^+/−^) mice^8^ were maintained on a C57BL/6J (B6) background. The R6/2 and HD190QG strains of HD model mice and the AR97Q strain of SBMA model mice were described previously^38^,^40^,^54^. R6/2 mice were maintained on a mixed background of B6/CBA. Repeat lengths of R6/2 mice were determined by GeneScan (Applied Biosystems) and transgenic animals with >120 repeats were used for experiments. For breeding of R6/2 mice, ovaries of R6/2 female mice with ~CAG130 were transplanted to B6CBAF1 female mice. Transplantation was carried out for 22 animals under pentobarbital anesthesia (40 mg/kg; Somnopentyl, Kyoritsu Seiyaku, Tokyo, Japan) using heating pad to maintain body temperature. The transplanted female mice were maintained at one animal per cage and crossed with B6CBAF1 male mice. When the average repeat size of the mouse population decreased to ~CAG120, R6/2 transgenic male mice were crossed with normal B6CBAF1 female mice to obtain animals with ~CAG130, of which the female mice were used for subsequent ovarian transplantation. Transplanted female mice were crossed with TLS^+/−^ male mice to generate HD-TLS^+/−^ mice and littermates. SBMA mice were maintained on the B6 background. SBMA-TLS^+/−^ mice were obtained by *in vitro* fertilization using the sperm of SBMA transgenic mice and the eggs of TLS^+/−^ mice. The CAG lengths of mutant AR were in the 85–96 range. For behavioral analysis, *in vitro* fertilization was performed to obtain large numbers of animals. The genotypes of all animals were confirmed by PCR. In all experiments, only male mice were used for all genotypes. Animals used for life span analysis were not used in behavioral or histological analyses. For the collection of tissues used for biochemical and transcriptome analyses, we used three or four animals for biochemical analysis and another three to four animals for RNA analysis, for each genotypes of HD-TLS and SBMA-TLS mice at 8 weeks, and 2 animals for HD190QG and non-transgenic mice at 24 weeks.

### Behavioral and life span analysis

The animal experimental room was maintained in 12 hr light-12 hr dark periods (light period: 8:00 AM to 8:00 PM). Each experiment was conducted by the same experimenter. Animals were tested blindly for genotype. We used euthanasia (pentobarbital anesthesia and cervical dislocation by trained staffs) for all animals at the end of the experiments or when animals suffered from the genetic or surgical interventions, except for those used in the life span analysis, which were approved by the institution’s Animal Ethics Committee.

For life span analysis, animals were maintained at five or less animals per cage with free access to water and food. Body weight of animals was recorded once a week. Mice were followed until their deaths in order to calculate their life spans.

A Rotarod test was performed using MK-610 (Muromachi Kikai Tokyo, Japan). The time duration of mice on the Rotarod was measured. In one set of experiments, three trials per animal were performed with intervals of 30 minutes between trials. For the analysis of SBMA-TLS mice, the Rotarod was used at fixed speed (20 rpm) for 3 minutes. The experiments were performed weekly starting from 4 weeks of age (n = 17 for SBMA-TLS^+/+^, n = 15 for SBMA-TLS^−/−^, n = 16 for WT-TLS^+/+^ and WT-TLS^+/−^). Since the number of surviving animals of SBMA-TLS^+/+^ and SBMA-TLS^+/−^ decreased considerably after 8 weeks old, we show the fraction of the animals with normal performance (Rotarod latency >120 sec) for older animals (Supplementary Fig. S3C). For the analysis of HD-TLS mice, an accelerating Rotarod was used, where the rotation was started with 4 rpm and gradually increased up to 40 rpm at 5 minutes and continued for 1 minute (n = 15 for WT-TLS^+/+^, n = 12 for WT-TLS^+/−^, n = 14 for HD-TLS^+/+^ and HD-TLS^+/−^). We analyzed the data using ANOVA followed by Tukey’s post-hoc test. Grip strength of four limbs was measured using a grip strength meter (MK-380, Muromachi Kikai, Tokyo, Japan), according to the manufacturer’s instructions. Mice were tested twice with an interval of at least 30 minutes and the larger value of the two trials was used. HD-TLS mice were analyzed at 6–8 weeks old (n = 14 for HD-TLS^+/+^ and HD-TLS^+/−^, n = 12 for WT-TLS^+/−^, and n = 15 for WT-TLS^+/+^).

For an open field test, an automatic detection system with nine 40 cm × 40 cm chambers was used for HD-TLS mice. Mouse behavior was analyzed for 30 minutes. Total distance of the movement, moving speed, and time spent in the center area were analyzed. HD-TLS mice were analyzed at 4, 6 and 8 weeks old (n = 14 for HD-TLS^+/−^, n = 16 for WT-TLS^+/+^ and WT-TLS^+/−^, and HD-TLS^+/+^).

### Identification of the components of Htt aggregates

A 24-week-old HD190QG or wild-type cerebrum was homogenized in RIPA buffer containing 1× protease inhibitor cocktail (Complete, EDTA free, Roche) at a ratio of 1.5 ml buffer per 0.1 mg tissue sample using Teflon homogenizers on ice. For preclearance, the lysate was reacted with protein A-conjugated magnetic beads (Dynabeads protein A) for 2 h at 4 °C at the ratio of 50 μl slurry of beads per 1 ml lysate. The beads was separated by magnetic holder and the supernatant was used for immunopurification. Anti-GFP (Molecular probe) was reacted with protein A-conjugated beads at room temperature for at least 2 h. We mixed the precleared lysates and the antibody-conjugated beads and incubated at 4 °C overnight with rotation. Then beads were separated from lysates, and washed with RIPA buffer at least three times. Following wash with PBS/0.1% Triton X-100 once, beads were treated with Benzonase nuclease (Merck Millipore) in PBS/0.1% Triton X-100 at 37 °C for 30 min. The solution was then separated from the beads and collected as the nuclease-sensitive fraction. We treated the beads with RIPA buffer containing 2% SDS at 37 °C for 1 h and collected the buffer as the SDS-sensitive fraction. After washing with RIPA/2% SDS once, formic acid was added to the beads and incubated at 37 °C for 3 h. The FA fraction was separated from the beads and subjected to a vacuum evaporator. After evaporation of FA, remaining proteins were re-solubilized by boiling in SDS sample buffer. Benzonase- and SDS-eluted fractions were also mixed with SDS sample buffer and boiled. The collected fractions were subjected to SDS-PAGE and CBB staining. Gel regions were excised, treated with iodoacetamide, and subjected to in-gel trypsin digestion (12.5 ng/ml) at 37 °C overnight. LC-MS/MS analysis was performed using a Paradigm MS2 HPLC pump (Michrom BioResources, Auburn, CA, USA) and LTQ linear ion trap mass spectrometer (Thermo Fisher Scientific, Inc., USA) equipped with nanospray ion source. The peptides were separated by InertSustain C18 (0.1 × 150 mm, GL Science) using a linear gradient (60 min, 5–65% acetonitrile/0.1% formic acid) at a flow rate of 500 nL/min. The resulting MS and MS/MS data were searched against the NCBInr database and EGFP sequence using MASCOT software (Matrix Science, United Kingdom). Peptides of TG mice with scores > = 40 (approximately corresponding to 95% confidence for many peptides) were used for searching the protein database. We selected proteins with Mascot scores of at least 60 as positive candidates. For negative control samples, peptides of WT mice with scores of at least 20 were used for database search to maximize the detection of false positives or contaminants. By comparing the results of WT and TG samples, we selected proteins specifically detected in TG samples as components of Htt aggregates, with the exception of histone proteins that were detected in the nuclease-sensitive fraction of WT proteins at much lower scores compared to those in TG proteins. Proteins detected at gel top of the SDS-sensitive fraction in SDS-PAGE were classified as SDS-resistant proteins. We excluded proteins that are highly likely to be contaminants such as trypsin, keratins, hemoglobin, and extracellular matrix proteins.

### Immunoprecipitation

We used Dynabeads Protein A (Veritas, Tokyo, Japan) for magnetic separation that can avoid centrifugation-mediated co-precipitation of non-specific proteins. For isolation of EGFP-fused Htt, anti-GFP antibody (598, MBL, Japan) was conjugated with Dynabeads at a ratio of 1 μl antibody to 30 μl slurry of the beads in PBS-0.01% Triton X-100 for 2 h at room temperature. For precipitation of FUS/TLS, anti-TLS-M was conjugated to the beads at a ratio of 1 μl antibody to 20 μl slurry of the beads. Typically, 5 μl of antibody per 1 ml brain lysate was used for immunoprecipitation. IP using anti-GFP was performed in RIPA buffer for an overnight at 4 °C, followed by wash with RIPA buffer 4–5 times. IP using anti-TLS-M was performed in a 1:1 mixture of RIPA and hypotonic buffer. Proteins bound to the beads were eluted with RIPA containing 1 M DTT and 2% SDS. All samples were mixed with SDS sample buffer and subjected to SDS-PAGE or AGERA.

### Agarose gel electrophoresis for resolving aggregates (AGERA)

Agarose gel preparation and electrophoresis were described previously^48^. For visualization of EGFP, the gel (with a glass plate on one side) was exposed to LAS4000 (GE Healthcare, Japan). For Western or mass analysis, we transferred the protein on a PVDF membrane using a conventional transfer apparatus for 90 minutes at 40 V, 4 °C or 150 mA at room temperature. Western analysis was performed as in the case of conventional SDS-PAGE analysis.

### Histological analysis and Western blot analysis

Antibodies used in this study are listed in Supplementary Information. Mouse section preparation and immunohistochemical analysis were performed as described^9^.

### cDNA clones and constructs

EGFP-fused N-terminal Htt constructs were described previously^55^. Truncated AR constructs were made by PCR amplification of a fragment corresponding to amino acids 1–132 from EGFP-AR constructs^56^ and subsequent insertion into pEGFP-C1 or pcDNA3 with mRFP^57^.

### Microarray analysis

Total RNA was extracted from frozen mouse striatum or spinal cord at 8 weeks of age using TRIzol reagent (Invitrogen). The resultant RNA was further purified using RNeasy and RNase-free DNase kit (Qiagen). 100 ng of RNA was amplified with Ambion WT Expression kit and labeled with WT Terminal Labeling kit (Affymetrix). Mouse Exon 1.0 ST array (Affymetrix) was hybridized with labeled probes according to the manufacture’s protocol. Dataset analysis was performed using GeneSpring GX (Agilent Technologies) and AltAnalyze^58^. For gene expression levels, raw P values <0.005 were considered significant. We noticed correction of multiple comparisons by the method of Benjamini and Hochberg was too stringent. For example, several genes known to be altered in HD (such as Penk, Hrh3, Nfya, and Polr2a) showed corrected P values larger than 0.05 in the ExonArray analysis of the comparison between HD-TLS^+/+^ and WT-TLS^+/+^, while the alteration of these genes were confirmed by qPCR analysis. Thus, exploration of altered genes using the corrected P-values might result in a large number of false negatives. For the same reason, we used raw P values for the evaluation of RNA processing analysis in AltAnalyze. We used the FIRMA method for detection of RNA processing. NCBI37/mm9 was used as a mouse genome assembly. DAVID^59^ was used for gene ontology analyses. Microarray data were deposited to Gene Expression Omnibus (GSE80004 and GSE80093).

### Quantitative PCR (qPCR)

Quantitative PCR analysis was performed as described previously^9^. In brief, we used LightCycler 480 and FastStart Universal SYBR Green Master (Roche Applied Science), according to the manufacturer’s protocol. Primer sets were designed using either PrimerExpress (Applied Biosystems) or PrimerQuest (Integrated DNA Technologies). We noticed that Gapdh expression was decreased in the striatum of HD-TLS^+/−^ mice by ~10% in both microarray and qPCR. We chose Cdk4 as a control for normalization in the analyses of HD-TLS crossed mice since it showed constant expression among different genotypes in ExonArray. Otherwise, Gapdh was used as a reference of normalization. For detection of human huntingtin exon 1 transgene, probe-based qPCR was performed using FastStart Universal Probe Master (Roche Applied Science). The fluorescent probe with double quenchers was purchased from Medical & Biological Laboratories. The sequences of primers are listed in Supplementary Information.

### Cell culture and transfection

Culture of Neuro2a (N2a) cells and transfection were performed as described^60^. For cell sorting, transfected cells in several 10 cm dishes were detached using trypsin and collected in DMEM. We sorted cells according to the fluorescence of EGFP using BD FACSAria. Collected cells (~5 × 10^5^ cells for each fraction) were frozen in liquid nitrogen. Cells were lysed in RIPA/2% SDS with sonication and the resulting total lysate was subjected to SDS-PAGE.

### Statistical analysis

We used a two-tailed unpaired t-test for comparison between two samples. We used ANOVA followed by Tukey’s post-hoc test for multiple comparison. For the analysis of survival rate, we used the Kaplan-Meier method followed by log-rank test. We considered the differences significant when P value is less than 0.05. In bar charts and line charts, error bars represent standard errors of the mean.

## Additional Information

**How to cite this article**: Kino, Y. *et al*. FUS/TLS acts as an aggregation-dependent modifier of polyglutamine disease model mice. *Sci. Rep*. **6**, 35236; doi: 10.1038/srep35236 (2016).

## Supplementary Material

Supplementary Information

Supplementary Table S1

Supplementary Table S2

Supplementary Table S3

## References

[b1] Kwiatkowski T. J. Jr. . Mutations in the FUS/TLS gene on chromosome 16 cause familial amyotrophic lateral sclerosis. Science 323, 1205–1208, doi: 10.1126/science.1166066 (2009). 19251627

[b2] Vance C. . Mutations in FUS, an RNA processing protein, cause familial amyotrophic lateral sclerosis type 6. Science 323, 1208–1211, doi: 10.1126/science.1165942 (2009). 19251628 PMC4516382

[b3] Deng H. X. . FUS-immunoreactive inclusions are a common feature in sporadic and non-SOD1 familial amyotrophic lateral sclerosis. Ann Neurol 67, 739–748, doi: 10.1002/ana.22051 (2010). 20517935 PMC4376270

[b4] Woulfe J., Gray D. A. & Mackenzie I. R. FUS-immunoreactive intranuclear inclusions in neurodegenerative disease. Brain Pathol 20, 589–597, doi: 10.1111/j.1750-3639.2009.00337.x (2010). 19832837 PMC8094734

[b5] Verbeeck C. . Expression of Fused in sarcoma mutations in mice recapitulates the neuropathology of FUS proteinopathies and provides insight into disease pathogenesis. Mol Neurodegener 7, 53, doi: 10.1186/1750-1326-7-53 (2012). 23046583 PMC3519790

[b6] Mitchell J. C. . Overexpression of human wild-type FUS causes progressive motor neuron degeneration in an age- and dose-dependent fashion. Acta Neuropathol 125, 273–288, doi: 10.1007/s00401-012-1043-z (2013). 22961620 PMC3549237

[b7] Qiu H. . ALS-associated mutation FUS-R521C causes DNA damage and RNA splicing defects. J Clin Invest 124, 981–999, doi: 10.1172/JCI72723 (2014). 24509083 PMC3938263

[b8] Hicks G. G. . Fus deficiency in mice results in defective B-lymphocyte development and activation, high levels of chromosomal instability and perinatal death. Nat Genet 24, 175–179, doi: 10.1038/72842 (2000). 10655065

[b9] Kino Y. . FUS/TLS deficiency causes behavioral and pathological abnormalities distinct from amyotrophic lateral sclerosis. Acta Neuropathol Commun 3, 24, doi: 10.1186/s40478-015-0202-6 (2015). 25907258 PMC4408580

[b10] Fujii R. & Takumi T. TLS facilitates transport of mRNA encoding an actin-stabilizing protein to dendritic spines. J Cell Sci 118, 5755–5765, doi: jcs.02692 (2005). 16317045 10.1242/jcs.02692

[b11] Ishigaki S. . Position-dependent FUS-RNA interactions regulate alternative splicing events and transcriptions. Sci Rep 2, 529, doi: 10.1038/srep00529 (2012). 22829983 PMC3402842

[b12] Lagier-Tourenne C. . Divergent roles of ALS-linked proteins FUS/TLS and TDP-43 intersect in processing long pre-mRNAs. Nat Neurosci 15, 1488–1497, doi: 10.1038/nn.3230 (2012). 23023293 PMC3586380

[b13] Rogelj B. . Widespread binding of FUS along nascent RNA regulates alternative splicing in the brain. Sci Rep 2, 603, doi: 10.1038/srep00603 (2012). 22934129 PMC3429604

[b14] Couthouis J. . A yeast functional screen predicts new candidate ALS disease genes. Proc Natl Acad Sci USA 108, 20881–20890, doi: 10.1073/pnas.1109434108 (2011). 22065782 PMC3248518

[b15] Kato M. . Cell-free formation of RNA granules: low complexity sequence domains form dynamic fibers within hydrogels. Cell 149, 753–767, doi: 10.1016/j.cell.2012.04.017 (2012). 22579281 PMC6347373

[b16] Kwon I. . Phosphorylation-regulated binding of RNA polymerase II to fibrous polymers of low-complexity domains. Cell 155, 1049–1060, doi: 10.1016/j.cell.2013.10.033 (2013). 24267890 PMC4010232

[b17] Doi H. . RNA-binding protein TLS is a major nuclear aggregate-interacting protein in huntingtin exon 1 with expanded polyglutamine-expressing cells. J Biol Chem 283, 6489–6500, doi: 10.1074/jbc.M705306200 (2008). 18167354

[b18] Bauer P. O. & Nukina N. The pathogenic mechanisms of polyglutamine diseases and current therapeutic strategies. J Neurochem 110, 1737–1765, doi: 10.1111/j.1471-4159.2009.06302.x (2009). 19650870

[b19] Woodman B. . The Hdh(Q150/Q150) knock-in mouse model of HD and the R6/2 exon 1 model develop comparable and widespread molecular phenotypes. Brain Res Bull 72, 83–97, doi: S0361-9230(06)00352-2 (2007). 17352931 10.1016/j.brainresbull.2006.11.004

[b20] Sathasivam K. . Identical oligomeric and fibrillar structures captured from the brains of R6/2 and knock-in mouse models of Huntington’s disease. Hum Mol Genet 19, 65–78, doi: 10.1093/hmg/ddp467 (2010). 19825844 PMC2792149

[b21] Kuhn A. . Mutant huntingtin’s effects on striatal gene expression in mice recapitulate changes observed in human Huntington’s disease brain and do not differ with mutant huntingtin length or wild-type huntingtin dosage. Hum Mol Genet 16, 1845–1861, doi: 10.1093/hmg/ddm133 (2007). 17519223

[b22] Landles C. . Proteolysis of mutant huntingtin produces an exon 1 fragment that accumulates as an aggregated protein in neuronal nuclei in Huntington disease. J Biol Chem 285, 8808–8823, doi: 10.1074/jbc.M109.075028 (2010). 20086007 PMC2838303

[b23] Sathasivam K. . Aberrant splicing of HTT generates the pathogenic exon 1 protein in Huntington disease. Proc Natl Acad Sci USA 110, 2366–2370, doi: 10.1073/pnas.1221891110 (2013). 23341618 PMC3568346

[b24] Hackam A. S. . The influence of huntingtin protein size on nuclear localization and cellular toxicity. J Cell Biol 141, 1097–1105 (1998). 9606203 10.1083/jcb.141.5.1097 PMC2137174

[b25] Saudou F., Finkbeiner S., Devys D. & Greenberg M. E. Huntingtin acts in the nucleus to induce apoptosis but death does not correlate with the formation of intranuclear inclusions. Cell 95, 55–66, doi: S0092-8674(00)81782-1 (1998). 9778247 10.1016/s0092-8674(00)81782-1

[b26] Arrasate M., Mitra S., Schweitzer E. S., Segal M. R. & Finkbeiner S. Inclusion body formation reduces levels of mutant huntingtin and the risk of neuronal death. Nature 431, 805–810, doi: nature02998 (2004). 15483602 10.1038/nature02998

[b27] Miller J. . Identifying polyglutamine protein species *in situ* that best predict neurodegeneration. Nat Chem Biol 7, 925–934, doi: 10.1038/nchembio.694 (2011). 22037470 PMC3271120

[b28] Baldo B. . TR-FRET-based duplex immunoassay reveals an inverse correlation of soluble and aggregated mutant huntingtin in huntington’s disease. Chem Biol 19, 264–275, doi: 10.1016/j.chembiol.2011.12.020 (2012). 22365609

[b29] Legleiter J. . Mutant huntingtin fragments form oligomers in a polyglutamine length-dependent manner *in vitro* and *in vivo*. J Biol Chem 285, 14777–14790, doi: 10.1074/jbc.M109.093708 (2010). 20220138 PMC2863238

[b30] Doi H. . Identification of ubiquitin-interacting proteins in purified polyglutamine aggregates. FEBS Lett 571, 171–176, doi: 10.1016/j.febslet.2004.06.077 (2004). 15280037

[b31] Nagaoka U. . Increased expression of p62 in expanded polyglutamine-expressing cells and its association with polyglutamine inclusions. J Neurochem 91, 57–68, doi: 10.1111/j.1471-4159.2004.02692.x (2004). 15379887

[b32] Deng H. X. . Mutations in UBQLN2 cause dominant X-linked juvenile and adult-onset ALS and ALS/dementia. Nature 477, 211–215, doi: 10.1038/nature10353 (2011). 21857683 PMC3169705

[b33] Couthouis J. . Evaluating the role of the FUS/TLS-related gene EWSR1 in amyotrophic lateral sclerosis. Hum Mol Genet 21, 2899–2911, doi: 10.1093/hmg/dds116 (2012). 22454397 PMC3373238

[b34] Mori F. . Optineurin immunoreactivity in neuronal nuclear inclusions of polyglutamine diseases (Huntington’s, DRPLA, SCA2, SCA3) and intranuclear inclusion body disease. Acta Neuropathol 123, 747–749, doi: 10.1007/s00401-012-0956-x (2012). 22318854

[b35] Korac J. . Ubiquitin-independent function of optineurin in autophagic clearance of protein aggregates. J Cell Sci 126, 580–592, doi: 10.1242/jcs.114926 (2013). 23178947 PMC3654196

[b36] Fujita K. . A functional deficiency of TERA/VCP/p97 contributes to impaired DNA repair in multiple polyglutamine diseases. Nat Commun 4, 1816, doi: 10.1038/ncomms2828 (2013). 23652004 PMC4543262

[b37] Elden A. C. . Ataxin-2 intermediate-length polyglutamine expansions are associated with increased risk for ALS. Nature 466, 1069–1075, doi: 10.1038/nature09320 (2010). 20740007 PMC2965417

[b38] Kotliarova S. . Decreased expression of hypothalamic neuropeptides in Huntington disease transgenic mice with expanded polyglutamine-EGFP fluorescent aggregates. J Neurochem 93, 641–653, doi: JNC3035 (2005). 15836623 10.1111/j.1471-4159.2005.03035.x

[b39] Weiss A. . Sensitive biochemical aggregate detection reveals aggregation onset before symptom development in cellular and murine models of Huntington’s disease. J Neurochem 104, 846–858, doi: JNC5032 (2008). 17986219 10.1111/j.1471-4159.2007.05032.x

[b40] Katsuno M. . Testosterone reduction prevents phenotypic expression in a transgenic mouse model of spinal and bulbar muscular atrophy. Neuron 35, 843–854, doi: S0896627302008346 (2002). 12372280 10.1016/s0896-6273(02)00834-6

[b41] Tallent M. K. . Cortistatin overexpression in transgenic mice produces deficits in synaptic plasticity and learning. Mol Cell Neurosci 30, 465–475, doi: S1044-7431(05)00186-7 (2005). 16182561 10.1016/j.mcn.2005.08.010 PMC13719228

[b42] Wang X. . Induced ncRNAs allosterically modify RNA-binding proteins in cis to inhibit transcription. Nature 454, 126–130, doi: 10.1038/nature06992 (2008). 18509338 PMC2823488

[b43] Sanchez-Ramos C. . PGC-1alpha regulates translocated in liposarcoma activity: role in oxidative stress gene expression. Antioxid Redox Signal 15, 325–337, doi: 10.1089/ars.2010.3643 (2011). 21338289

[b44] Wang W. Y. . Interaction of FUS and HDAC1 regulates DNA damage response and repair in neurons. Nat Neurosci 16, 1383–1391, doi: 10.1038/nn.3514 (2013). 24036913 PMC5564396

[b45] Hoell J. I. . RNA targets of wild-type and mutant FET family proteins. Nat Struct Mol Biol 18, 1428–1431, doi: 10.1038/nsmb.2163 (2011). 22081015 PMC3230689

[b46] Fratta P. . FUS is not dysregulated by the spinal bulbar muscular atrophy androgen receptor polyglutamine repeat expansion. Neurobiol Aging 34, 1516, e1517–e1519, doi: 10.1016/j.neurobiolaging.2012.09.008 (2013). 23062703

[b47] Doi H. . p62/SQSTM1 differentially removes the toxic mutant androgen receptor via autophagy and inclusion formation in a spinal and bulbar muscular atrophy mouse model. J Neurosci 33, 7710–7727, doi: 10.1523/JNEUROSCI.3021-12.2013 (2013). 23637164 PMC6618982

[b48] Kurosawa M. . Depletion of p62 reduces nuclear inclusions and paradoxically ameliorates disease phenotypes in Huntington’s model mice. Hum Mol Genet 24, 1092–1105, doi: 10.1093/hmg/ddu522 (2015). 25305080

[b49] Han T. W. . Cell-free formation of RNA granules: bound RNAs identify features and components of cellular assemblies. Cell 149, 768–779, doi: 10.1016/j.cell.2012.04.016 (2012). 22579282

[b50] Kim H. J. . Mutations in prion-like domains in hnRNPA2B1 and hnRNPA1 cause multisystem proteinopathy and ALS. Nature 495, 467–473, doi: 10.1038/nature11922 (2013). 23455423 PMC3756911

[b51] Verma A. & Tandan R. RNA quality control and protein aggregates in amyotrophic lateral sclerosis: a review. Muscle Nerve 47, 330–338, doi: 10.1002/mus.23673 (2013). 23381726

[b52] Safren N. . Ubiquilin-1 overexpression increases the lifespan and delays accumulation of Huntingtin aggregates in the R6/2 mouse model of Huntington’s disease. PLoS One 9, e87513, doi: 10.1371/journal.pone.0087513 (2014). 24475300 PMC3903676

[b53] Ishiura H. . The TRK-fused gene is mutated in hereditary motor and sensory neuropathy with proximal dominant involvement. Am J Hum Genet 91, 320–329, doi: 10.1016/j.ajhg.2012.07.014 (2012). 22883144 PMC3415534

[b54] Mangiarini L. . Exon 1 of the HD gene with an expanded CAG repeat is sufficient to cause a progressive neurological phenotype in transgenic mice. Cell 87, 493–506, doi: S0092-8674(00)81369-0 (1996). 8898202 10.1016/s0092-8674(00)81369-0

[b55] Kino Y. . Intracellular localization and splicing regulation of FUS/TLS are variably affected by amyotrophic lateral sclerosis-linked mutations. Nucleic Acids Res 39, 2781–2798, doi: 10.1093/nar/gkq1162 (2011). 21109527 PMC3074126

[b56] Bauer P. O. . Harnessing chaperone-mediated autophagy for the selective degradation of mutant huntingtin protein. Nat Biotechnol 28, 256–263, doi: 10.1038/nbt.1608 (2010). 20190739

[b57] Kino Y. . MBNL and CELF proteins regulate alternative splicing of the skeletal muscle chloride channel CLCN1. Nucleic Acids Res 37, 6477–6490, doi: 10.1093/nar/gkp681 (2009). 19720736 PMC2770659

[b58] Emig D. . AltAnalyze and DomainGraph: analyzing and visualizing exon expression data. Nucleic Acids Res 38, W755–W762, doi: 10.1093/nar/gkq405 (2010). 20513647 PMC2896198

[b59] Huang da W., Sherman B. T. & Lempicki R. A. Systematic and integrative analysis of large gene lists using DAVID bioinformatics resources. Nat Protoc 4, 44–57, doi: 10.1038/nprot.2008.211 (2009). 19131956

[b60] Kino Y. . Nuclear localization of MBNL1: splicing-mediated autoregulation and repression of repeat-derived aberrant proteins. Hum Mol Genet 24, 740–756, doi: 10.1093/hmg/ddu492 (2015). 25274774

