# Supplementary material for: FUS/TLS acts as an aggregation-dependent modifier of polyglutamine disease model mice
Source: Sci Rep. 2016 Oct 14;6:35236. doi: 10.1038/srep35236 (PMC5064419; doi:10.1038/srep35236)
Supplement: Supplementary Information [file srep35236-s1.pdf]

Supplemental Information

FUS/TLS acts as an aggregation-dependent modifier of polyglutamine disease model mice.

Yoshihiro Kino, Chika Washizu, Masaru Kurosawa, Mizuki Yamada, Hiroshi Doi, Toru Takumi, Hiroaki Adachi, Masahisa Katsuno, Gen Sobue, Geoffrey G. Hicks, Nobutaka Hattori, Tomomi Shimogori, and Nobuyuki Nukina.

Supplementary Methods

Antibodies

Primary antibodies used in this study are listed bellow. Rabbit anti-TLS-M, anti-TLS-C, anti-EWS-M, anti-TAF15-M, and anti-Uqbln2 were described previously (Doi et al., 2004; Doi et al., 2008). Secondary antibodies were anti-mouse, anti-rabbit, anti-goat, or anti-Guinea pig IgG antibodies conjugated to Alexa-488, Alexa-546, or Alexa-634 (Molecular Probe), to horse radish peroxidase (ECL anti-rabbit or anti-mouse IgG Horseradish Peroxidase Linked whole antibody, GE Healthcare, and Peroxidase-conjugated AffiniPure Donkey Anti-Goat IgG, Jackson Immuno research), or to biotin (Vector Laboratories).

Antibody	source	number	host
TLS-M	Doi et al. 2008	-	rabbit
TLS-C	Doi et al. 2008	-	rabbit
EWS-M	Doi et al. 2008	-	rabbit
TAF15-M	Doi et al. 2008	-	rabbit
UBQLN2	Doi et al. 2004	-	rabbit
hnRNP A1 (9H10) :	Santa Cruz	sc-56700	mouse
NMDAR1 (NR1-CT)	upstate	06-311	rabbit
PSD95	Abcam	ab18258	rabbit
DDX5	Abcam	ab10261	goat
alpha-tubulin	Sigma	T9026	mouse
gamma-tubulin (C-20)	Santa Cruz	sc-7396	goat
Htt (EM48)	Millipore	MAB5374	mouse
HSC70 (K-19)	Santa Cruz	sc-1059	goat
Sqstm1	Progen	GP62-C	Guinea pig
Sqstm1	MBL	PM045	rabbit
TDP43	Proteintech Group	BC001487 10782-1	rabbit
Htt 2B4 (polyP)	Millipore	MAB5492	mouse
TLS 4H11	Santa Cruz	sc-47711	mouse
GFP	Molecular Probe	A6455	rabbit

GFP	Roche	11 814 460 001	mouse
GFP	MBL	598	Rabbit
DDDDK-tag (Flag)	MBL	M185-3L	mouse
AR (N-20)	Santa Cruz	sc-816	rabbit
AR (H-280)	Santa Cruz	sc-13062	rabbit
AR	Everest	EB06441	goat
polyglutamine (1C2)	Chemicon	MAB1574	mouse
LaminB (M-20)	Santa Cruz	sc-6217	goat
AAK1	Abcam	Ab173329	rabbit
TRK-fused (TFG)	Abcam	Ab150428	rabbit
CBP	Sigma	SAB4500455	rabbit
FUS/TLS	Sigma	HPA008784	rabbit
Rad23b (C-4)	Santa Cruz	sc-166507	mouse
Ubiquitin	Chemicon	MAB1510	mouse

Quantitative PCR (qPCR)

The sequences of primers are listed as follows.

Name	sequence (5'-3')	Name	sequence (5'-3')
RT-Tls-3UTR-Fw	CCCAGTGTTACCCTTGTTATTTTGT	RT-Ews-Fw	CAATCCGGGCTGTGGAAA
RT-Tls-3UTR-Rv	CCCCCCAAAAAAAAATATCCA	RT-Ews-Rv	GCCTTACACTGGTTGCATTCTG
RT-hAR-Fw	GCTGCACCGACCGTGAGT	RT-Rad9b-Fw	TGTATTTCCAAGACAGCCAGCCCT
RT-hAR-Rv	GTTCGTGTCGCCGGCC	RT-Rad9b-Rv	TGTCAGAAGAGCAATGGCCTCAGT
RT-hHtt-ex1-Fw	GCTGCACCGACCGTGAGT	RT-Casp9-Fw	TCTTCACGCGCGACATGA
RT-hHtt-ex1-Rv	GTTCGTGTCGCCGGCC	RT-Casp9-Rv	CCGCCGAGACCCAGATC
RT-Htt-int1-probe	FAM-CAGCTCCCTGTCCCGGCGG-ZEN/IBFQ	RT-Myo7a-Fw	CGAGGGATTCAGCCTTTTTG
RT-Gdpd3-Fw	GGGTCAGACCGGCACATG	RT-Myo7a-Rv	CAGTCTGTCAGGTGTCGGACAA
RT-Gdpd3-Rv	CATGGGAGTCCTTGGAAATTTC	RT-Gabra2-Fw	GCCGAATGTCCCATGCA
RT-Cort-Fw	TGTCCAAGAGCCAGGAAAGG	RT-Gabra2-Rv	GGGCATGAATGAGCATCCAT
RT-Cort-Rv	TCTTGCAGGGCTTTTTATCCA	RT-Igfals-Fw	GGGCCTCAGTCACCTTTGG
RT-Miip-Fw	AGAGCAGCGGTGTGGAAGA	RT-Igfals-Rv	CGTGTCAGGCAGGACCACTA
RT-Miip-Rv	GCCGGCGGTTGATACG	RT-Zfp69-Fw	TGTCAGTGGCAGGATATCAACTTT
RT-Arnt2-Fw	TGGTCCACTGCACAGGTTACA	RT-Zfp69-Rv	GGTCCCTCTCCTTTTTCCAACT
RT-Arnt2-Rv	TTCTTCGGGTATGGTCATTCCT	RT-Masp2-Fw	CGCCTGCGCCTCTACTTC

RT-Hrh3-Fw	GCCACTGCTATGCTGAGTTCTTCT	RT-Masp2-Rv	TCATACTCGCAGCGGTAAGAGA
RT-Hrh3-Rv	AAGAAGGTAACGCTGAGGAAGGGT	RT-Gm7609-Fw	CAAGGAATGCGGGCCTTAC
RT-Taf15-Fw	TCAAAACCAACAGTCTTACCATTCA	RT-Gm7609-Rv	CCCCAAATACCAGCCAACAC
RT-Taf15-Rv	CACGACGGTCATCTTGTGTGT		
RT-Hnrnpa1-Fw	GGGTTTGTCACATATGCCACTGT		
RT-Hnrnpa1-Rv	CCACCTTGTGTGGTCTTGCA		

Biochemical analysis of mouse tissues

Total lysates were prepared as described [9]. All extraction buffers were supplemented with 1× complete protease inhibitor cocktail (Roche) and pre-chilled on ice, except for those containing 2% SDS. For fractionation analysis shown in Supplementary Fig. S1B and S1C, cerebrum of 24-week-old wild-type or transgenic HD190QG mice were homogenized in hypotonic buffer [10 mM HEPES (pH7.9), 1.5 mM $MgCl_2$, 10 mM KCl]. NP-40 was added to the homogenate to a final concentration of 0.5 % and incubated on ice for 30 minutes. The homogenate was centrifuged at 20000×g for 15 minutes. The supernatant was collected and designated as Cyto. The pellet was suspended in PBS and treated with Benzonase nuclease (Merck Millipore) at 4°C for 3 h. The mixture was centrifuged at 20000×g for 15 minutes and the supernatant was collected and designated as S1. Nuclear extraction buffer (NE) [20mM HEPES (pH7.9), 1.5 mM $MgCl_2$, 0.42M NaCl, 25% glycerol] was added to the pellet fraction and incubated on ice for 30 minutes with brief vortex mixing every 10 minutes. The mixture was centrifuged at 20000×g for 15 minutes and the supernatant was collected as NE1. By repeating NE extraction twice more, we obtained the resultant supernatants designated as NE2 and NE3. Then, the pellet was washed once with NE, which was collected as Wash (W). To the pellet, RIPA buffer [50 mM Tris-HCl (pH 8.0), 150 mM NaCl, 1% Triton X-100, 0.5% sodium deoxycholate, 1mM EDTA (pH 8.0)] was added and briefly sonicated using Branson Sonifier for 10 seconds three times at the setting of

7 on ice. The mixture was centrifuged at 20000×g for 15 minutes and the supernatant was collected and designated as S2. RIPA supplemented with 2% SDS (RIPA/2%SDS) was added to the pellet and sonicated for 10 seconds three times at room temperature. The mixture was centrifuged at 20000×g for 15 minutes and the supernatant was collected as S3. The remaining pellet was suspended in RIPA/2% SDS by sonication and designated as Ppt. The samples were subjected to AGERA.

Immunoprecipitation using Flag-TLS

Flag-tagged FUS/TLS was made by inserting the murine TLS coding sequence amplified by PCR into the NotI-XbaI sites of p3xFlag-CMV-7.1 (Sigma-Aldrich). EGFP-AR(tr)23Q and EGFP-AR(tr)99Q were co-transfected with Flag-TLS into the N2a cells. 48hours post-transfection, cells were harvested and subjected to immunoprecipitation using anti-Flag-M2 agarose affinity gel (Sigma-Aldrich) in RIPA buffer. After washed with RIPA containing 500mM NaCl five times, immunoprecipitates were eluded by boiling in the 1x SDS sample buffer and subjected to Western blot analysis.

Supplementary Tables (See separate Excel file)

Table S1. Protein components of Htt aggregates identified from mass analysis.

Proteins were eluted from immunoprecipitates of HD190QG transgenic mice and were subjected to LC-MS/MS analysis. Identified proteins are listed. Proteins with a Mascot score of at least 60 were selected. "Matches" indicate the total number of matched peptides including overlaps.

Table S2. Gene expression analysis of HD and SBMA mice.

Gene expression changes detected using ExonArray and AltAnalyze. Genes that were altered 1.2-fold at a raw P value of <0.005 are listed.

Table S3: RNA processing analysis of HD and SBMA mice.

RNA processing changes detected using ExonArray and AltAnalyze. Exons that were altered by FIRMA fold change >2.0 at a raw P value of <0.005 are listed. Spread sheet modified from AltAnalyze.

Supplementary Figure S1

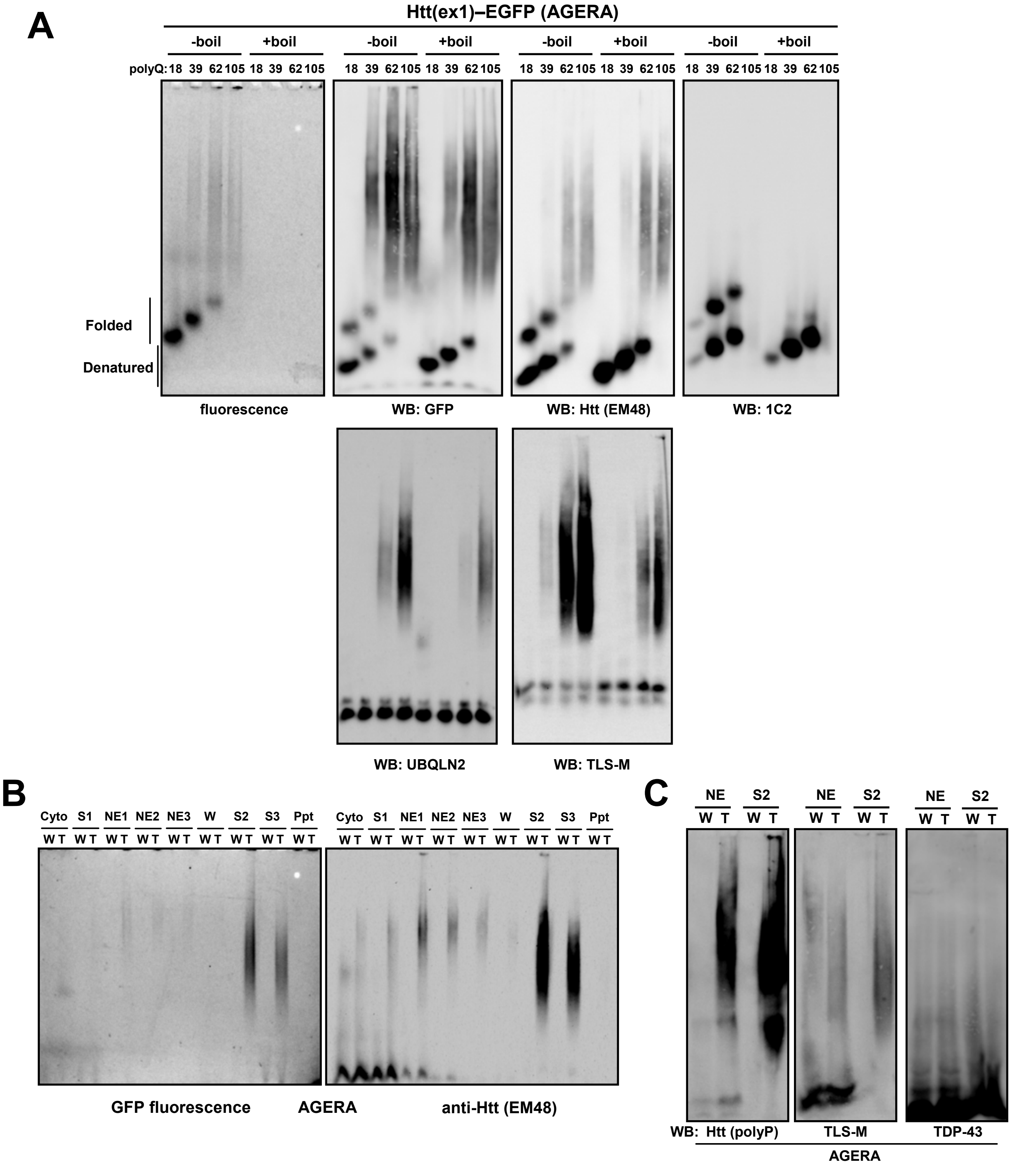

Figure S1. Agarose gel analysis of Htt aggregates in N2a cells and HD190QG mice.

(A) Agarose gel analysis of aggregates (AGERA) of Htt exon1 fused with EGFP with different polyQ lengths. 2% SDS-containing total lysate of N2a cells expressing indicated proteins with or without heat treatment were subjected to agarose gel electrophoresis. The fluorescence of EGFP was monitored. Then, the proteins were transferred onto a PVDF membrane and detected by indicated antibodies. EGFP fluorescence was at least in part preserved in 2% SDS-containing buffer but completely eliminated by heat treatment, which also altered migration pattern of monomeric proteins. FUS/TLS co-migrated with GFP-contining aggregates even after heat treatment. While anti-GFP and EM48 stained smear of aggregates, 1C2 did not stained these protein populations, suggesting that polyQ tract of aggregates are inaccessible because of protein-protein interactions. **(B)** AGERA of HD190QG-derived fractions. EGFP fluorescence (left panel) and immunostaining (right panel) are shown. W: wild type and T: transgenic animals. **(C)** AGERA of NE and S2 fractions from HD190QG and control mice stained with Htt, TLS-M or TDP43 antibody.

Supplementary Figure S2

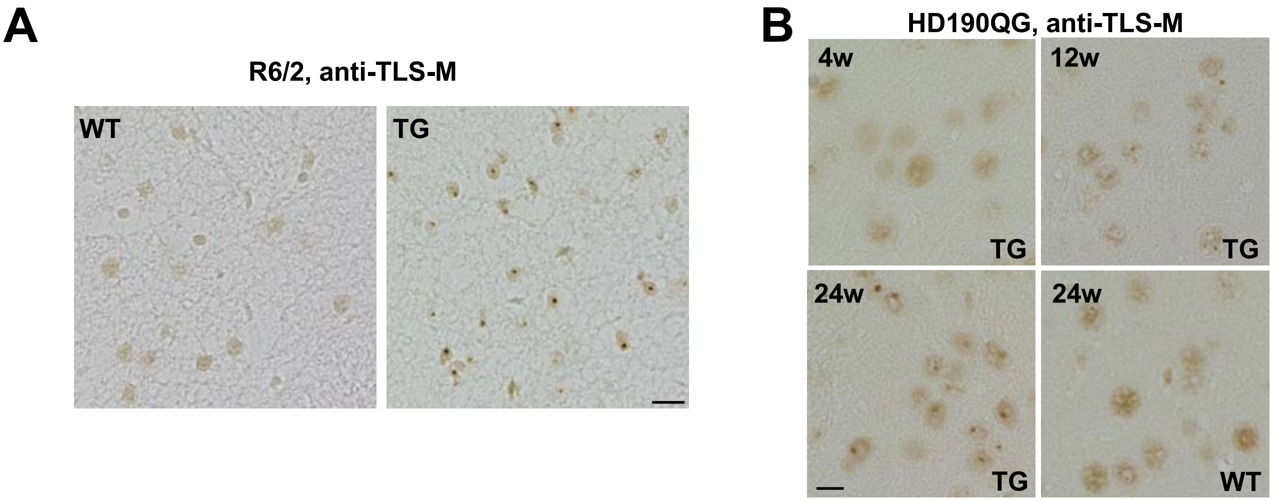

Figure S2. Immunohistochemical analysis of HD model mice.

(A) FUS/TLS-positive inclusions in the R6/2 striatum. Sections from a wild type (WT) mouse and a transgenic (TG) mouse were stained with anti-TLS-M antibody. Scale bar: 10 μm. **(B)** Staining of FUS/TLS in the striatal sections of HD190QG mice using anti-TLS-M. Scale bar: 10 μm.

Supplementary Figure S3

A

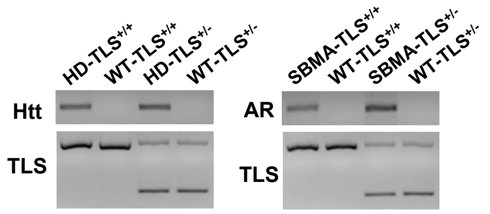

B

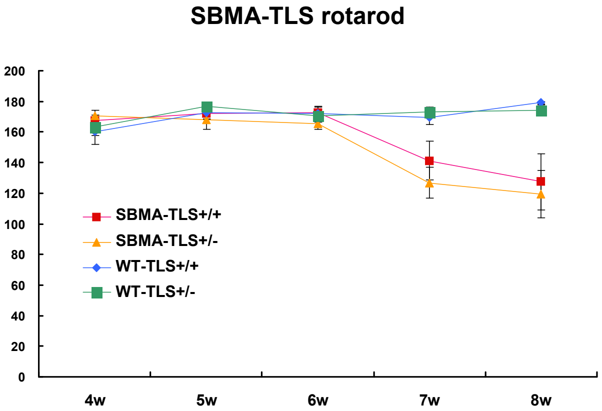

C

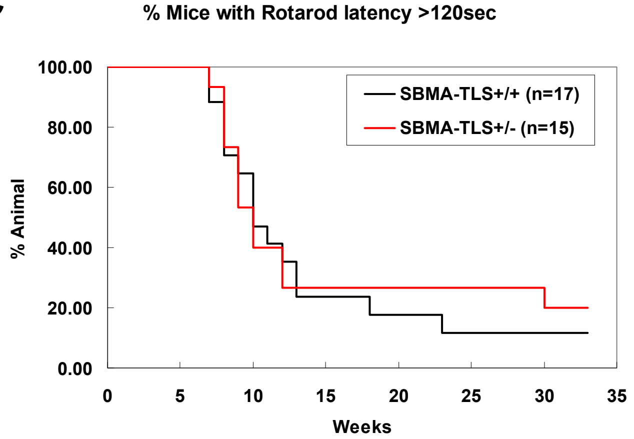

D

Effects of heterozygous depletion in HD models

Modified gene	Genotype	Pathway	Model	Life span	Motor function	Author (year)
CBP (Crebbp)	+/-	Transcription	N171	39% ↓	No effect	Klevytska (2010)
FUS/TLS	+/-	RNA processing	R6/2	23.6% ↓	Worsened	This study
Hsf2	+/-	Transcription	R6/2	17.2 % ↓	n.a.	Shinkawa (2011)
PGC-1alpha (Ppargc1a)	+/-	Transcription	KI	n.a.	Worsened	Cui (2006)
BDNF	+/-	Growth factor	R6/1	n.a.	Worsened	Canals (2004)
Hsp70.1/70.3	+/-	Chaperone	R6/2	No effect	Worsened	Wacker (2009)
Parkin (PARK2)	+/-	UPS, mitochondria	R6/1	n.a.	n.a.	Rubio (2009)
SIRT2	+/-	Energy metabolism	R6/2	n.a.	No effect	Bobrowska (2012)
UBC	+/-	Ubiquitin	R6/2	n.a.	No effect	Bett (2009)
Complexin II	+/-	Presynapse	R6/2	No effect	No effect	Glynn (2007)
tissue transglutaminase	+/-	Protein modification	R6/2	No effect	No effect	Bailey (2004)
Cyclophilin D	+/-	Mitochondria	R6/2	No effect	No effect	Perry (2010)
HDAC3	+/-	Histone modification	R6/2	n.a.	No effect	Mounme (2012)
HDAC7	+/-	Histone modification	R6/2	n.a.	No effect	Benn (2009)
alpha-synuclein	+/-	Parkinson's disease	R6/1	No effect	Improved	Tomás-Zapico (2012)
Rhes	+/-	mTOR, SUMO	R6/1	n.a.	Improved	Baiamonte (2013)
Caspase-6	+/-	Apoptosis	BACHD	n.a.	Improved	Gafni (2012)
Sp1	+/-	Transcription	R6/2	8.4% ↑	n.a.	Qiu (2006)
IRS2	+/-:Tg*	Energy metabolism	R6/2	42% ↑	Improved	Sadagurski (2011)

n.a.: not analyzed

*pancreatic beta-cell specific rescue of IRS2

Figure S3. Rotarod analysis of SBMA-TLS mice and a summary of genetic interaction of HD model mice.

(**A**) PCR genotyping of animals obtained by genetic crossing. (**B**) Rotarod analysis of SBMA-TLS crossed mice. SBMA mice showed reduced performance regardless of FUS/TLS genotypes. Mean ± SEM, n=17 for SBMA-TLS$^{+/+}$, n=15 for SBMA-TLS$^{+/-}$, n=16 for WT-TLS$^{+/+}$ and WT-TLS$^{+/-}$. (**C**) Fraction of mice showing rotarod performance with >120 seconds on the rod at different time points. (**D**) Summary of HD mouse genetic experiments using heterozygous knockout animals. KI: knock-in.

Supplementary Figure S4

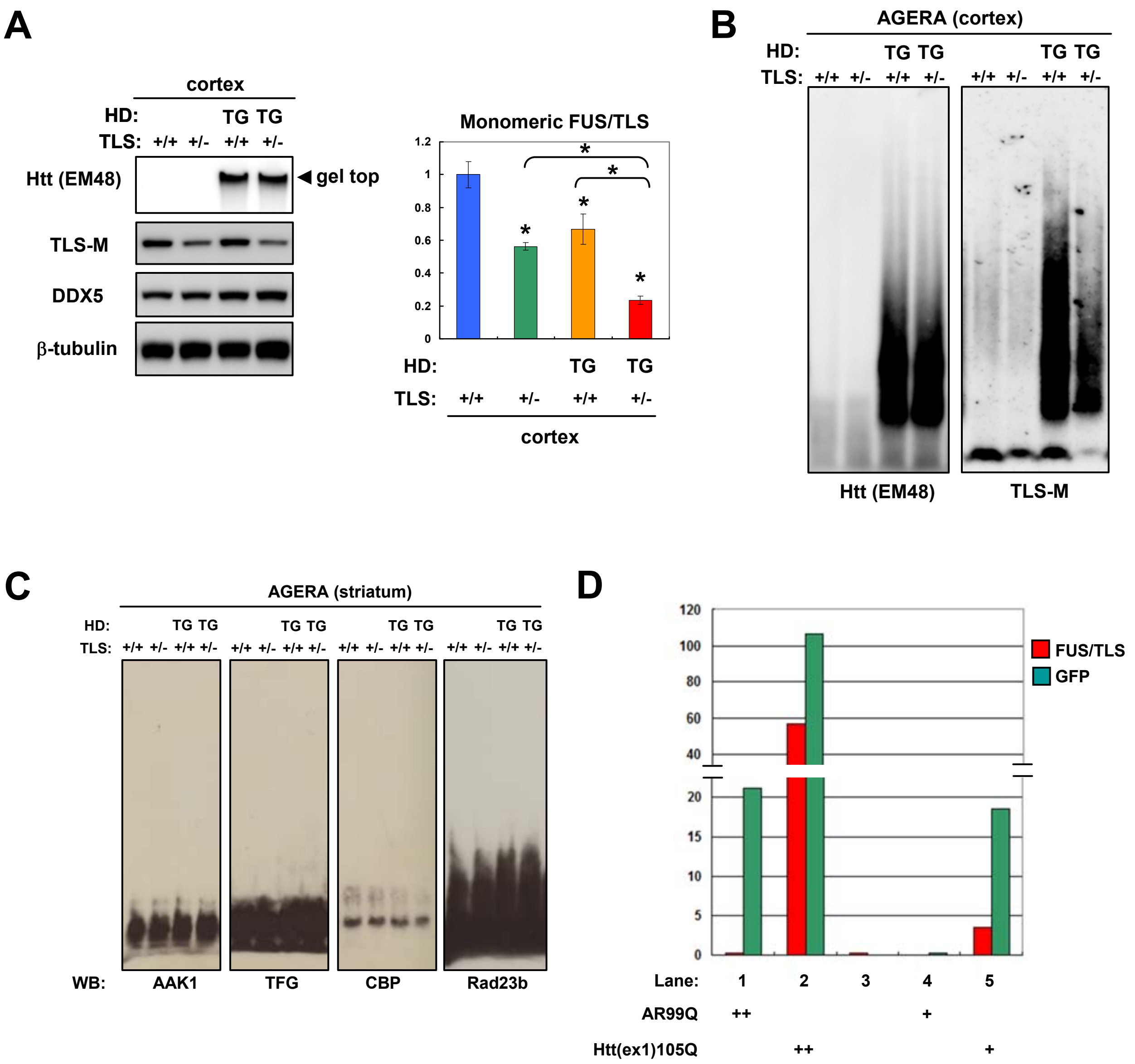

Figure S4. Protein analysis of Htt aggregates in HD-TLS crossed mice.

(**A**) SDS-PAGE analysis of cerebral cortex samples from 8-week-old TLS-HD crossed mice (left). Quantified results of the amount of monomeric FUS/TLS normalized by DDX5 (right). Bars represent mean ± SEM (n=3). *P<0.05 in comparison with WT-TLS$^{+/+}$ or indicated comparisons by a Tukey's test (n=3). (**B**) AGERA analysis of cerebral cortex samples from 8-week-old TLS-HD crossed mice. Western blot was performed using antibodies indicated. (**C**) AGERA analysis of striatum samples from 8-week-old TLS-HD crossed mice. Western blot was performed using antibodies indicated. (**D**) Quantification of proteins at the gel-top aggregates in Fig. 4B. Bars indicate protein amounts determined by the Western blot densitometry at the gel top (arbitrary unit).

Supplementary Figure S5

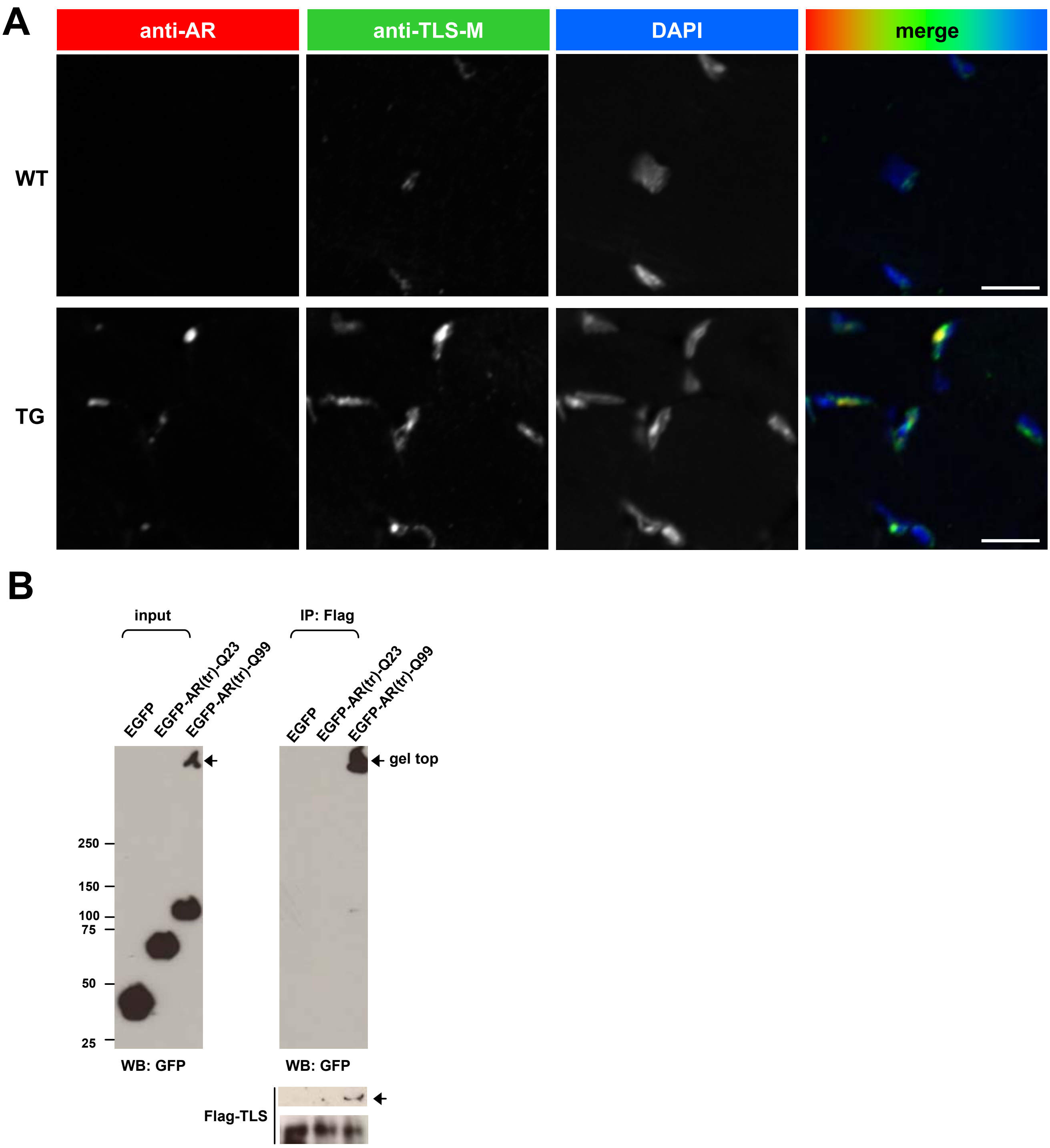

Figure S5. Interaction between mutant AR and FUS/TLS.

(A) Colocalization of FUS/TLS and AR inclusions in the skeletal muscle of SBMA model mice at 8 weeks. Sections of wild type (WT) and transgenic (TG) mice were stained with anti-TLS-M antibody (green) and goat anti-AR antibody (red). Nuclei were stained with DAPI. Representative confocal images are shown. Scale bar = 10 μm. **(B)** Co-immunoprecipitation of EGFP-fused AR and Flag-tagged FUS/TLS in N2a cells. EGFP-AR fragments were co-transfected with Flag-TLS. Cell lysate of transfected cells were subjected to immunoprecipitation using anti-Flag antibody. We detected aggregated EGFP-AR(tr)-99Q in the eluted fraction. Arrows indicate the gel top.

Supplementary Figure S6

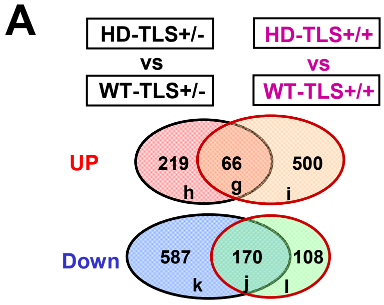

	Term	Count	%	P-Value	corrected P
g	no term with corrected P value <0.05	-	-	-	-
h	no term with corrected P value <0.05	-	-	-	-
i	acetylation	85	18	4.3E-06	1.2E-03
	protein transport	25	5.3	1.2E-04	1.7E-02
	alternative splicing	132	28	2.8E-04	2.0E-02
	phosphoprotein	176	37.3	2.4E-04	2.2E-02
j	membrane	89	53.3	1.7E-11	3.0E-09
	plasma membrane	59	35.3	3.1E-10	4.6E-08
	cell membrane	40	24	1.4E-08	1.2E-06
	nucleoside-triphosphatase regulator activity	19	11.4	1.0E-08	1.5E-06
	GTPase regulator activity	19	11.4	7.8E-09	2.2E-06
	dendrite	11	6.6	7.2E-08	5.4E-06
	metal ion transmembrane transporter activit	16	9.6	8.7E-08	6.2E-06
	cation channel activity	15	9	8.1E-08	7.7E-06
	ion channel activity	17	10.2	1.6E-07	9.2E-06
	substrate specific channel activity	17	10.2	2.5E-07	1.2E-05
k	ionic channel	33	5.9	6.9E-11	2.4E-08
	cation channel activity	30	5.4	1.4E-10	7.9E-08
	metal ion transmembrane transporter activit	32	5.7	3.1E-10	9.0E-08
	voltage-gated cation channel activity	21	3.8	6.7E-10	9.7E-08
	gated channel activity	31	5.6	5.7E-10	1.1E-07
	voltage-gated channel	21	3.8	7.6E-10	1.3E-07
	ion channel activity	34	6.1	1.9E-09	2.2E-07
	membrane	215	38.5	1.9E-09	2.2E-07
	substrate specific channel activity	34	6.1	4.2E-09	4.0E-07
	channel activity	34	6.1	5.9E-09	4.9E-07
l	sterol biosynthesis	5	5.4	1.9E-06	2.6E-04
	sterol metabolic process	7	7.6	5.4E-07	3.2E-04
	Steroid biosynthesis	5	5.4	1.6E-05	1.0E-03
	cholesterol metabolic process	6	6.5	7.8E-06	1.6E-03
	sterol biosynthetic process	5	5.4	5.8E-06	1.7E-03
	Cholesterol biosynthesis	4	4.3	4.1E-05	1.8E-03
	steroid metabolic process	7	7.6	3.9E-05	5.9E-03
	cholesterol biosynthetic process	4	4.3	1.0E-04	1.2E-02
	steroid biosynthetic process	5	5.4	1.8E-04	1.8E-02
	lipid synthesis	5	5.4	5.4E-04	1.8E-02

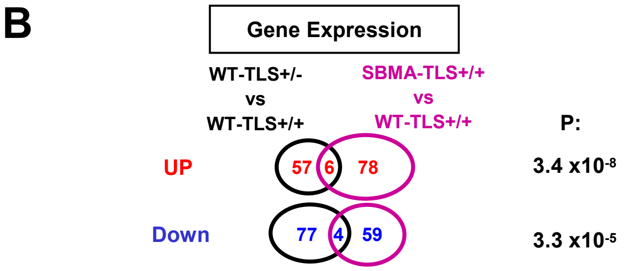

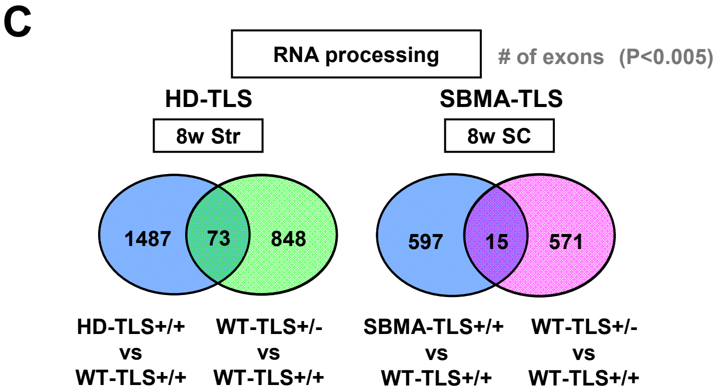

Figure S6. Transcriptome analysis of HD-TLS and SBMA-TLS mice.

(A) Comparison of genes altered in the striatum between HD-TLS$^{+/-}$ and WT-TLS$^{+/-}$ and those altered between HD-TLS$^{+/+}$ and WT-TLS$^{+/+}$. Venn diagrams show the number of genes. Lowercase letters indicate groups of genes in the diagram. Mean ± SEM (n=3). Bottom list shows the results of gene ontology analysis of gene groups using DAVID. Terms with a corrected P value <0.05 are shown. **(B)** Overlaps of genes differentially regulated in the spinal cord of SBMA-TLS mice (raw P value <0.005, 1.2 fold). Comparisons were conducted as in Figure 4C. **(C)** Overlaps of exons differentially regulated in WT-TLS$^{+/+}$ vs WT-TLS$^{+/-}$ and HD(SBMA)-TLS$^{+/+}$ vs WT-TLS$^{+/+}$ (raw P value <0.005 in AltAnalyze). Str: striatum, SC: spinal cord.
